# Impact of physical exercise on the regulation of brain-derived neurotrophic factor in people with neurodegenerative diseases

**DOI:** 10.3389/fneur.2024.1505879

**Published:** 2025-01-28

**Authors:** Ana Romero Garavito, Valery Díaz Martínez, Estherz Juárez Cortés, José Vicente Negrete Díaz, Liliana Marcela Montilla Rodríguez

**Affiliations:** ^1^Facultad de medicina, Universidad Cooperativa de Colombia, Villavicencio, Colombia; ^2^Programa de Fisioterapia, Universidad de Guanajuato, Guanajuato, Mexico; ^3^Programa de Psicologia Clinica, Universidad de Guanajuato, Guanajuato, Mexico

**Keywords:** physical exercise, brain-derived neurotrophic factor (BDNF), neurodegenerative diseases, dementia, Parkinson’s disease, neuroplasticity

## Abstract

This review explores the impact of physical exercise on brain-derived neurotrophic factor (BDNF) and its relationship with neurodegenerative diseases. The key role of BDNF in maintaining brain health is highlighted, and recent studies are analyzed that indicate an increase in BDNF levels following physical activity, particularly in young adults. Additionally, the interaction between the BDNF Val66Met genetic polymorphism and exercise on cognitive function is examined. The review emphasizes the possibility of exercise as a complementary therapy for neurodegenerative diseases, although further research is required to fully understand its effects.

## 1 Introduction

According to data from the World Health Organization (WHO), neurodegenerative diseases and mental disorders represent a significant public health concern worldwide. It is estimated that more than 55 million people suffer from dementia, with Alzheimer’s disease being the most common form (1). Around 30% of individuals worldwide have encountered a mental disorder at some stage in their lives (2), and neurological conditions were responsible for more than 9 million fatalities in 2016 alone (3). Additionally, the occurrence of Parkinson’s disease has risen substantially in recent years. In 2019, it was estimated that over 8.5 million people were impacted, effectively doubling the figures reported 25 years earlier (4).

Brain-derived neurotrophic factor (BDNF) is vital for the survival, maintenance, and regeneration of specific neuronal populations in the adult central nervous system. Its role is critical in supporting neuronal health and facilitating neuroplasticity. Secreted by neurons and glial cells, brain-derived neurotrophic factor (BDNF) primarily facilitates neuronal survival, supports synaptic plasticity, and encourages neurogenesis. BDNF plays a significant role in various brain functions, such as memory, learning, and emotional regulation. Furthermore, it has been linked to the protection and recovery of the nervous system following injury or in the context of neurodegenerative diseases (5).

The decline in neurotrophic factor levels, particularly brain-derived neurotrophic factor (BDNF) and its receptors, is a well-documented physiological event in neurodegenerative conditions like Alzheimer’s disease (6, 7), Parkinson’s disease (8, 9), multiple sclerosis (10, 11) and amyotrophic lateral sclerosis (12). BDNF plays a neuroprotective role, particularly in the hippocampus, where it helps mitigate the effects of neurodegeneration. Consequently, a reduction in BDNF plasma levels is closely linked to deteriorating brain health (13, 14).

Exercise has been shown to offer considerable advantages for both muscular and metabolic disorders, and there has been a growing focus on its effects on cognitive function. It is crucial to note that these effects necessitate comprehensive research due to their potential positive influence on various conditions, including neurodegenerative diseases and psychiatric disorders. The acknowledgment of these benefits has resulted in the consideration of physical exercise as a supplementary therapeutic approach in both research and clinical settings (3). Thus, investigating the relationship between brain-derived neurotrophic factor and physical activity is vital for preventing the onset of multiple pathologies and enhancing the pathophysiological characteristics of various diseases.

This review examines the impact of physical exercise on the regulation of brain-derived neurotrophic factor (BDNF) in individuals with neurodegenerative diseases, focusing on brain plasticity and neuroprotection. While previous studies have linked exercise to the release of BDNF, aspects such as the influence of different types of exercise (aerobic, resistance, strength) on BDNF modulation in diseases like Alzheimer’s and Parkinson’s have not been explored in depth. A better understanding of how exercise regimens affect BDNF regulation in these patients is needed, as neurodegenerative diseases could alter this response. The novelty of this review lies in its integrated approach, which not only examines how exercise influences BDNF levels but also explores the molecular mechanisms involved in neuroplasticity, neurogenesis, and cognitive improvement. Additionally, the therapeutic implications of increasing BDNF levels through physical exercise in patients with neurodegenerative diseases are highlighted, an area that remains underexplored. This opens new avenues for non-pharmacological therapeutic strategies to complement conventional treatments. In summary, this review aims to establish a clearer connection between the molecular mechanisms underlying BDNF-mediated neuroplasticity and how physical exercise may support these processes in the context of neurodegeneration. It is expected that this analysis will provide new insights into the development of more accessible and effective therapeutic interventions for neurodegenerative disorders.

## 2 Methodology

This review synthesized information on the relationship between physical exercise, brain-derived neurotrophic factor (BDNF), and molecular mechanisms linked to neuroplasticity and neurodegenerative diseases. Data collection occurred using databases such as PubMed, Google Scholar, ScienceDirect, and EBSCOhost and MEDLINE. Search terms combined: brain-derived neurotrophic factor (BDNF), Physical exercise, neurodegenerative diseases, dementia, Parkinson’s disease, neuroplasticity. The selection process involved inclusion and exclusion criteria emphasizing experimental and non-experimental studies focusing on exercise, BDNF, and neuroplasticity-related variables. Abstracts were screened for relevance, followed by full-text evaluation to confirm study alignment with the review’s objectives. Inclusion criteria included studies conducted in humans or rodents, published in English or Spanish, and examining exercise as an independent variable affecting BDNF, or long-term potentiation. Exclusion criteria included studies lacking pre-and post-exercise measurements, those offering incentives for cognitive testing, or failing to analyze exercise as a primary factor. Selected articles underwent manual duplication checks, forming the basis for the analysis and sub-sections of the review.

## 3 Brain-derived neurotrophic factor (BDNF)

Brain-Derived Neurotrophic Factor (BDNF), sometimes referred to as abrineurina, is part of the neurotrophic growth factor family (15). BDNF is first synthesized as the pro-BDNF isoform, with a molecular weight ranging from 28 k to 32 kDa, the canonical form being 28 kDa. This pro-BDNF is cleaved both intracellularly and extracellularly to produce the mature BDNF (mBDNF) of 14 kDa and a 17 kDa propeptide. Pro-BDNF interacts with the p75NTR receptor, while mBDNF binds to the TrkB receptor, triggering signaling pathways that support neuronal survival. In contrast, signaling through the p75NTR receptor by pro-BDNF can induce apoptotic or pruning effects on neurons, indicating a nuanced relationship between the two isoforms (16).

BDNF is synthesized presynaptically in the cell bodies of sensory neurons that project to the dorsal horn, whereas in the hippocampus, its primary source is postsynaptic dendrites (17, 18). Peripherally, BDNF is detected in serum and is associated with blood plasma platelets, as well as being present in vascular endothelial cells and peripheral blood mononuclear cells (19, 20). Within the peripheral nervous system, BDNF also contributes to axonal regeneration. The expression of the BDNF gene and its receptor, TrkB, is found in various other tissues throughout the body, including endothelial cells, lungs and the heart (21, 22). BDNF is highly expressed in the hippocampus, a brain region known for its plasticity and critical involvement in learning and memory processes (23, 24) This neurotrophic factor forms a complex with its primary receptor, tropomyosin receptor kinase B (TrkB), which initiates a cascade of signaling pathways vital for neuronal function (14). BDNF has been implicated in various processes, including cognitive modulation, neuroplasticity, angiogenesis, and synaptogenesis (25, 26). Research has demonstrated that levels of BDNF can significantly impact both structural and functional changes in the brain (27), influencing hippocampal neurogenesis (25), long-term potentiation (28), increases in hippocampal volume, and the survival of hippocampal neurons (29, 30). Furthermore, the deterioration of the hippocampus has been strongly associated with multiple neurodegenerative diseases (31–34).

### 3.1 BDNF genetics

The Val66Met single nucleotide polymorphism (SNP), also referred to as rs6265, is the most prevalent genetic modifier of BDNF. This specific polymorphism results in the substitution of valine for methionine at codon 66 (35). Research involving various animal models has demonstrated that replacing one or two Val alleles with a Met allele leads to a decrease in exercise-induced BDNF expression within the hippocampus, which subsequently impairs hippocampal plasticity and memory formation (36).

While these findings have not yet been definitively validated in humans, evaluations of hippocampal memory functions in individuals carrying Val and Met alleles suggest that similar effects may occur in humans as well (37). Furthermore, several previous genomic studies have indicated a correlation between BDNF levels and the Val66Met polymorphism; however, not all investigations have consistently supported this relationship (38, 39).

## 4 Effects of physical exercise on BDNF levels and its relationship with brain health

Physical exercise is an essential factor for reducing morbidity and mortality, as well as improving quality of life in individuals with chronic diseases. It has been shown to be effective both in the prevention and management of these conditions. However, most patients do not engage in sufficient physical activity, and global healthcare systems offer few supervised programs, limiting the implementation of sustainable strategies for promoting an active lifestyle (40–43). Recognized by the American College of Sports Medicine (ACSM), physical exercise provides significant benefits in the prevention and treatment of diseases, particularly through its effects on the brain and nervous system (44, 45). In older adults (55–80 years), activities such as walking on a treadmill at moderate intensity three times a week have been shown to increase hippocampal volume by 2%, improving spatial memory and strengthening neural networks (26). Additionally, this type of exercise strengthens communication within neural networks, enhancing memory (46). Furthermore, angiogenesis and the proliferation of endothelial cells have been observed in the cerebral cortex and hippocampus (44, 45, 47).

Table 1 summarizes how various modalities, intensities, and durations of physical exercise interact with biological, genetic, and population factors to modulate BDNF levels. Evidence highlights that high-intensity exercises and sustained regimens are the most effective in promoting brain health, cognitive function, and psychological well-being, mediated by interactions between BDNF, its receptor TrkB, and neuronal plasticity.

**Table 1:** Effects of physical exercise on BDNF levels and its relationship with brain health.

Influencing factor	Scientific description	Scientific evidence and study relationships	References
Exercise intensity and type	Physical exercise increases peripheral BDNF levels, with the most significant effects seen in aerobic and high-intensity exercises.	Independent studies show that HIIT and aerobic exercise promote benefits in neuroplasticity and cognitive function (15, 176–179).	(15, 180, 181)
Exercise duration	Both acute and prolonged exercise increase BDNF, but the effect is more sustained with regular, long-term regimens.	A meta-analysis found more significant BDNF increases in prolonged studies involving women and older adults. Acute studies show immediate effects.	(54, 182)
High-intensity (HIIT)	Increases BDNF, cortisol, and lactate, promoting metabolic and psychological adaptations.	García-Suárez et al. and Fernández et al. agree that HIIT elicits more pronounced BDNF responses compared to moderate-intensity exercise.	(175, 176, 181)
Aerobic and resistance training	Improves cardiovascular and brain function, positively influencing neuroplasticity.	Prolonged aerobic exercise increases BDNF and improves vascular and cognitive functions, with positive effects observed in older adults.	(183, 184)
Dose–response model	The impact of exercise on BDNF expression is proportional to the intensity and frequency of the stimulus.	In animal models, forced and voluntary exercise increased hippocampal BDNF, improving spatial memory and synaptic function.	(164, 185, 186)
Animal models	Increases in BDNF and its receptors (TrkB) are associated with improved synaptic plasticity and cognition.	In Ts65Dn mice, aerobic exercise enhanced spatial memory and discrimination. Blocking TrkB receptors reduced these benefits.	(160, 187)
Genetic polymorphisms (SNPs)	BDNF Val66Met polymorphisms modulate exercise responses regarding BDNF levels and cognitive performance.	Piepmeier et al. observed that vigorous exercise increased mBDNF, but the response was lower in carriers of the Val66Met allele (81, 188).	(69, 189)
Sex and age	Exercise responses vary by sex and age, being more pronounced in older adults and men.	Bugge et al. found higher serum BDNF levels in men. Women showed diurnal fluctuations associated with the hypothalamic–pituitary–adrenal axis.	(188, 189)
Physical and emotional well-being	Regular exercise improves mood, reduces depressive and anxious symptoms, and optimizes neuroplasticity.	Studies in women associated elevated BDNF levels with better psychological conditions following HIIT and military training programs.	(87, 190, 191)

Studies in humans and animals demonstrate that the effects of exercise are not only immediate but also cumulative, particularly in vulnerable populations such as older adults or individuals with genetic predispositions. These findings suggest a therapeutic role for exercise in neurodegenerative and mental health conditions, emphasizing the importance of designing personalized interventions based on individual characteristics such as sex, age, and BDNF polymorphisms.

## 5 BDNF as a mediator of neurogenesis and neuronal plasticity

Neuroplasticity refers to the nervous system’s capacity to adjust to environmental changes, which includes modifications in the number of neurons and the enhancement of synaptic connections. This mechanism is essential for learning and memory, enabling organisms to respond appropriately to various environmental stimuli and challenges. At the cellular level, one of the most studied mechanisms related to neuroplasticity and underlying memory formation is long-term potentiation (LTP) (48). Both the performance of exercise and the subsequent increases in BDNF facilitate more efficient and enduring neuronal changes, leading to improvements in long-term potentiation (LTP) (49).

The positive effects of consistent physical activity on neuroplasticity and cognitive functions are clear, particularly through the stimulation of BDNF production. This increase in BDNF fosters improved neuronal development, enhances learning and memory capabilities, and helps prevent cognitive decline. Thus, exercise not only supports general well-being but also significantly contributes to the enhancement of cognitive processes and neuronal plasticity (50). In this regard, BDNF has been recognized as an essential mediator in central synaptic plasticity, a process fundamental for memory formation and learning. It has been observed that BDNF can influence both the facilitation and induction of synaptic changes by stabilizing preexisting synapses and promoting the establishment of new connections (51).

Furthermore, research has shown that BDNF plays a pivotal role in enhancing synaptic transmission, leading to sustained increases like those seen in long-term potentiation (LTP) within brain regions such as the hippocampus and neocortex. This underscores BDNF’s essential function in modulating both synaptic structure and function, positioning it as a significant regulator of cognitive processes (52). By activating its receptor, tropomyosin receptor kinase B (TrkB), BDNF not only promotes neuronal survival but also enhances synaptic plasticity. Conversely, its precursor, proBDNF, has an opposing role; it induces cellular apoptosis through its interaction with the p75 neurotrophin receptor (p75NTR), illustrating their divergent physiological functions (17, 53). Accordingly, the binding of BDNF to TrkB is integral to the mechanisms that facilitate the cognitive enhancements associated with exercise, particularly in learning and memory (54, 55). In contrast, it has been suggested that irisin, a protein released during muscular activity, might increase BDNF expression at the mRNA level and its corresponding protein synthesis. However, the direct connection between BDNF, irisin, and memory in humans is not yet fully established due to the lack of concurrent analyses. Both human studies and animal models have employed various experimental strategies to investigate this association. It has been observed that exercise enhances cognitive processes such as memory through changes in LTP. However, when TrkB receptors, which are essential for BDNF action, are inactivated during exercise, these cognitive effects are not greater than those in control groups (14, 56).

### 5.1 Neuroplastic changes induced by BDNF expression

Physical activity can promote neuroplastic changes by modulating the expression of proteins such as BDNF, which is linked to neuronal growth, survival, and synaptic plasticity. Both epidemiological and intervention studies lend support to the notion of utilizing physical exercise to enhance neuroplasticity, especially in pathological contexts. Research has shown that exercise not only leads to structural modifications in the brain but also provides protection against cognitive decline associated with aging (57, 58). Furthermore, more significant cognitive improvements are observed when exercise programs are longer in duration, when participants are older, and when comparing benefits for women to those for men, with women experiencing greater advantages (59). Notably, these exercise-related benefits are also evident in young adult men; a cohort study involving young Swedish men who enlisted in military service at age 18 revealed a substantial positive correlation between cardiovascular fitness and cognitive performance (60).

Several studies explore the differences between free BDNF levels in plasma and serum, with a particular focus on the origin and implications of these differences. According to Radka et al., BDNF levels in human and rat serum are significantly higher than in plasma, with plasma BDNF levels being up to 50 times lower. This BDNF in plasma appears to be derived from platelet degranulation, as it correlates positively with serotonin levels, a marker of platelet release. In contrast, mouse serum did not contain detectable BDNF, reflecting an interspecies difference in the expression of BDNF in specific brain tissues, highlighting its uneven distribution in the nervous system (61) On the other hand, Yoshimura et al. observed that serum BDNF levels are approximately 14 times higher than plasma levels, suggesting that serum, due to its platelet content, contains a higher amount of circulating BDNF. This is also supported by studies showing strong correlations between plasma and serum BDNF levels in healthy volunteers, supporting the idea that differences in BDNF concentration between the two compartments are due to platelet release (62). In a clinical context, Silva et al. reviewed studies on depression and blood BDNF levels, highlighting that BDNF levels in the serum and plasma of depressed patients may reflect alterations in neurotrophic function associated with the pathology, and that these differences may be linked to suicide attempts. Studies also suggest that platelet BDNF content could be a more stable measure of BDNF levels than plasma measurements, due to the shorter half-life of BDNF in plasma (63).

A study examined how physical exercise (PE) and cognitive exercise (CE), both individually and in combination, affect brain function through neurobiological mechanisms that stimulate neuroplasticity. The research included 97 older adults aged 65 to 75, who were randomly divided into four groups: CE followed by rest, PE followed by rest, CE followed by PE, and PE followed by CE. Measurements of plasma BDNF (pBDNF) and serum BDNF (sBDNF) levels, as well as cognitive performance, were conducted both prior to and following the interventions. Results showed that pBDNF levels rose after CE, PE, and rest, while sBDNF levels increased exclusively after PE. Acute PE was found to elevate both pBDNF and sBDNF levels, but prolonged PE did not have the same effect; in contrast, CE only influenced pBDNF levels. No changes in cardiorespiratory fitness were detected in any of the groups. Initial levels of pBDNF and sBDNF were correlated but responded differently to exercise. These results suggest that the biological mechanisms underlying pBDNF and sBDNF responses to exercise require further investigation (64).

In 2020, Muller and his team examined the influence of lactate and BDNF on neuroplasticity resulting from exercise. They discovered that both lactate and BDNF are key mediators in this process. Lactate appears to act as a metabolic signal that triggers the release of BDNF during exercise, thereby promoting synaptic plasticity and neuronal survival (28). The interaction of BDNF with its receptor TrkB is involved in the processes that lead to enhancements in learning and memory associated with exercise (26, 51, 54, 55). In 2019, Lauretta El Hayek and her research team studied the impact of lactate on the influence of exercise on learning and memory using animal models. They studied how lactate activates the SIRT1 signaling pathway in the hippocampus, which in turn activates BDNF and improves learning and memory (65).

The advantages of exercise for BDNF production and neuronal plasticity are significantly associated with improved vascularization in both cerebral and muscular tissues. A recent review indicated that enhanced cardiovascular fitness yields cognitive benefits, which stem from increased cerebral circulation and angiogenesis. This adaptation promotes greater blood flow and an upregulation of neurotrophins within the hippocampus, even following acute exercise sessions (66). Furthermore, several researchers have suggested that the cognitive enhancements resulting from physical exercise may not solely be due to heightened blood perfusion in the brain; rather, they may also arise from the elevated expression of BDNF within the hippocampal structure (67, 68).

In 2019, Piepmeier and colleagues conducted preliminary research examining how varying intensities of acute exercise affect memory and concentrations of BDNF isoforms (proBDNF and mBDNF). The study also considered the influence of the BDNF val66met SNP polymorphism. Participants were divided into three groups: light-intensity exercise, vigorous-intensity exercise, and a control group with no exercise, with evaluations made based on their BDNF allele status. The results showed that exercise intensity and BDNF allele status differentially affect BDNF isoform concentrations and memory performance. Vigorous-intensity exercise increased mBDNF, while carriers of the BDNF allele exhibited lower concentrations of mBDNF. Light-intensity exercise improved memory, but BDNF allele carriers had worse memory performance at 24 h (69).

### 5.2 Molecular mechanism by which BDNF intervenes in plasticity

Physical exercise triggers a cascade of molecular and cellular mechanisms that foster neuronal plasticity and neurogenesis by promoting the expression of the BDNF-encoding gene (70).

#### 5.2.1 The BDNF/TrkB signaling pathway

Aerobic exercise is a form of physical activity that depends on the metabolic utilization of oxygen from the bloodstream for muscle function. BDNF, by interacting with a specific receptor on the cell surface known as tropomyosin receptor kinase B (TrkB), can initiate excitatory or inhibitory signaling pathways, leading to neuronal survival, growth, and differentiation (24, 26). The activation of TrkB triggered by BDNF can initiate distinct signaling pathways that are either excitatory or inhibitory, depending on the target neurons (71). In excitatory neurons, BDNF enhances the expression of molecules like VGluT2, which are critical for glutamatergic neurotransmission, thereby amplifying synaptic excitation (72, 73). Conversely, in inhibitory neurons, BDNF increases the expression of markers associated with GABAergic neurotransmission, such as GABA, GAD65, and GAD67 (74). However, this process is also linked to a reduction in potassium-chloride cotransporter 2 (KCC2) levels, which can disrupt inhibitory balance and, under certain conditions, diminish inhibitory function. The outcome of BDNF–TrkB activation depends on the neurotransmitter profile of the target synapses and the associated molecular changes, including VGluT2 and KCC2 expression (71).

Studies evaluating the cognitive abilities of rats exposed to different experimental conditions (enriched environment with toys and stimulating activities, while others underwent moderate exercise, such as running on a rotating wheel) demonstrate that the combination of moderate exercise and an enriched environment leads to a marked enhancement in learning and memory in rats, along with elevated levels of BDNF and TrkB expression in their brains (75).

Physical activity promotes hippocampal BDNF expression and TrkB signaling via lactate, a metabolite linked to enhanced learning and memory. This process involves FNDC5 (Fibronectin type III domain-containing protein 5), a myokine regulated by PGC1a and ERRa transcriptional activity. Despite uncertainties about how cleaved FNDC5 activates BDNF, these findings highlight a critical mechanism by which exercise benefits the central nervous system, offering potential therapeutic strategies for neurological disorders (65).

The relationship between BDNF, glutamate release, and calcium permeability is critical for synaptic plasticity. BDNF released from the postsynaptic neuron can act on both the same neuron and the presynaptic neuron. In the presynaptic neuron, BDNF increases the number of vesicles containing glutamate, thereby enhancing its release into the synapse (76). In the postsynaptic neuron, BDNF activation of the TrkB receptor boosts the activity of both AMPA and NMDA-type glutamate receptors, which increases their presence on the cell membrane. The activation of NMDA receptors allows a greater influx of Ca^2+^ into the cell, a process essential for long-term potentiation (LTP). This increase in calcium permeability, facilitated by glutamate release and receptor activity, underpins neuroplasticity in key brain regions, including the hippocampus and cerebral cortex, and supports neuronal survival, growth, and synaptic function (77). Furthermore, BDNF has been demonstrated to increase the density of TrkB receptors on the cell membrane, thereby supporting neuronal survival, growth, and synaptic plasticity in areas such as the hippocampus and cerebral cortex (78). Thus, the interplay between BDNF, glutamate release, and calcium influx is central to the mechanisms of synaptic plasticity.

#### 5.2.2 CREB pathway

All neurotrophins are first produced as prepro-neurotrophins and subsequently undergo structural modifications via proteolysis. Exercise can activate the CREB pathway, stimulating mRNA synthesis and protein production (79). BDNF, for example, is synthesized as a prepro-neurotrophin and stored in vesicles, where it is converted into pro-BDNF or its mature form, mBDNF, through proteolysis (80). These forms of BDNF have different effects when binding to their receptors, promoting neuroplasticity processes. Pro-BDNF, when binding to the p75NTR receptor, can induce apoptosis and synaptic depression, whereas mBDNF, when binding to TrkB receptors, activates enzymes that release more BDNF, enhancing neuroplasticity (81).

#### 5.2.3 Influence of physical exercise on BDNF and cognitive function

Table 2 summarizes the key factors influencing the relationship between physical exercise, Brain-Derived Neurotrophic Factor (BDNF), and cognitive function. It compiles the scientific descriptions of how exercise impacts BDNF expression and cognitive health, along with evidence from studies that elucidate the molecular, genetic, and aging-related dynamics. The table also explores the variability in exercise responses due to genetic factors, particularly the BDNF gene polymorphism, and highlights the molecular pathways involved in neuroprotection, including the roles of BDNF receptors and intracellular signaling. Additionally, it provides an overview of the findings regarding the effects of exercise on cognitive decline, aging, and neuroplasticity. The references cited are critical studies that support these insights.

**Table 2:** Influence of physical exercise on BDNF and cognitive function.

Influencing factor	Scientific description	Scientific evidence and study relationships	References
Neuroplasticity	Increases in BDNF facilitate neurogenesis, synaptogenesis, and memory consolidation.	In humans, regular exercise increases BDNF release from the brain to the periphery, improving cognition. Animal models support these findings.	(177, 178, 192, 193)
Physical Exercise and Cognitive Function	Physical exercise, especially aerobic, improves cognitive function and memory while attenuating hippocampal atrophy in older adults, with BDNF playing a key role.	Studies in humans and rodents have demonstrated that regular exercise elevates BDNF expression in the hippocampus, correlating with improvements in memory and cognitive abilities.	(54, 164, 176, 179, 181–183, 185, 186, 194)
Physical Exercise and BDNF Expression	In cognitively healthy individuals, BDNF levels in cerebrospinal fluid decrease with age, and low concentrations are associated with memory deficits and reduced cognitive abilities.	Longitudinal studies have shown that a six-month training program increased serum BDNF levels and improved cognitive function, also increasing hippocampal volume in older adults.	(26, 178, 182, 195)
BDNF and Cognitive Decline in Hypoxia	Despite increased BDNF levels from exercise, some studies suggest that elevated BDNF does not always prevent cognitive decline in certain conditions.	Piotrowicz et al. (196) found that athletes, despite increased BDNF levels from exercise, experienced cognitive decline following moderate hypoxia exposure, suggesting that BDNF may not protect in these conditions.	(196)
Genetic Variability and Exercise Response	The Val66Met polymorphism in the BDNF gene may modulate the impact of exercise on cognition.	Liu et al. (197) found heterogeneous results regarding the influence of this polymorphism but suggested that regular exercise might influence cognition in individuals with this polymorphism.	(197)
Multicomponent Exercise and Cognitive Function	Multicomponent exercise, including aerobic components, shows overall cognitive benefits, although its specific effects on cognitive impairment in dementia remain unclear.	Studies show that multicomponent exercise, with an aerobic component, improves cognitive function, though its effect on mild cognitive impairment and dementia remains uncertain.	(169, 198)
Molecular Mechanisms in Exercise and Cognition	Exercise increases neurogenesis, neurotransmitter synthesis, and the release of neurotrophic factors such as BDNF, which may enhance cognitive functions.	Montero et al. (56) discussed the association of exercise with cognitive improvements, including memory and attention, and molecular mechanisms such as BDNF release.	(56, 174, 195)
BDNF and TrkB Signaling Pathways	BDNF interacts with its high-affinity receptor, TrkB, activating key intracellular pathways involved in neuroprotection induced by exercise.	Studies in animal models show that consistent exercise increases BDNF and TrkB expression. This effect can be inhibited by BDNF antisense oligonucleotides, suggesting a critical role in exercise-induced neuroprotection.	(160, 164, 185–187, 195)
P2X4R and P2X7R Receptors in Exercise	P2X4R and P2X7R receptors play an essential role in modulating the neuroprotective effects of BDNF in response to exercise.	Research has demonstrated that these receptors are critical in modulating BDNF activity and neuroprotection, providing insights into neuronal health and function.	(199)
Aging and BDNF in Exercise	The hippocampus is particularly vulnerable to aging, and aerobic exercise may mitigate age-related atrophy by increasing BDNF expression.	Studies on aging have shown results regarding BDNF levels. Long-term athletic training has been associated with improvements in brain health, reducing oxidative damage and increasing BDNF levels. BDNF is likely an indicator closely linked to cognitive performance.	(20, 187, 200–205)

## 6 BDNF and exercise in neurodegenerative diseases and neuropsychiatric disorders

Brain-derived neurotrophic factor (BDNF) is essential in various neuropsychiatric disorders (82). Lower BDNF levels have been associated with neurodegenerative conditions, including Alzheimer’s and Parkinson’s diseases, leading to declines in memory and cognitive function (83). Conversely, higher BDNF levels in the brain correlate with reduced cognitive decline and enhanced memory and recall abilities (84, 85). Furthermore, BDNF is linked to genetic networks related to brain aging and Alzheimer’s disease (38, 86). Research has shown that physical exercise can significantly elevate plasma BDNF levels in individuals with neurodegenerative disorders (87). BDNF exerts its effects on neurons through various intracellular signaling pathways activated by TrkB or p75 receptors. Clinically, BDNF has been implicated in the pathophysiology of multiple brain diseases (30).

In 2022, Campbell and colleagues examined the impact of early-life stress on the regulation of BDNF and explored how exercise interventions may influence this relationship. The study examined the effects of early-life stress in animal models and evaluated how exercise could modulate changes in BDNF expression. The results indicated that early-life stress can negatively impact BDNF regulation, potentially contributing to mental and cognitive health issues. However, exercise interventions demonstrated potential for counteracting these negative effects by increasing BDNF expression and promoting neuronal plasticity in the brain. In summary, the study indicates that physical activity could serve as a beneficial intervention to counteract the negative effects of early-life stress on BDNF regulation, potentially offering important benefits for mental health and emotional well-being across the lifespan (88).

### 6.1 Genetic interaction of the Val66Met polymorphism with environmental factors in neurodegenerative diseases and neuropsychiatric disorders

Multiple studies have established a connection between the Val66Met polymorphism and neuropsychiatric disorders; however, most of these findings have not been replicable, possibly due to interactions among factors such as age, sex, environment, and ethnic origin (5). Evidence indicates that both genetic and environmental factors impact brain development and the risk of neuropsychiatric disorders. Among the environmental factors, including adverse prenatal environments, childhood trauma, climate, and life stress, are crucial in the development of these brain disorders (89, 90).

The BDNF Val66Met polymorphism interacts with multiple environmental and epigenetic factors, heightening the likelihood of developing neuropsychiatric disorders. Specifically, this polymorphism has been linked to amnestic mild cognitive impairment and its advancement to Alzheimer’s disease, attributed to increased methylation of the BDNF promoter (91). For example, in older women with anxiety/depression, increased DNA methylation of BDNF was found, particularly in BDNF Val66Met heterozygotes (92). Conversely, in the context of depression, the BDNF Val66Met polymorphism influences the association between life stress and depression, with a more pronounced effect observed for life stress events compared to childhood adversity (93).

Serum levels of brain-derived neurotrophic factor (BDNF) are influenced by factors such as age, physical activity, and neuropsychiatric disorders, including dementia, depression, anxiety, schizophrenia, and bipolar disorder. A study examining military personnel revealed an increased risk of mental health disorders within this population. This research specifically assessed serum BDNF levels among military samples, encompassing administrative staff and three groups from the Special Operations Forces (SOF). Notably, the SOF-TC group demonstrated higher BDNF levels compared to the control group, which was not subjected to stress. This elevation may be attributed to variations in age or the effects of rigorous physical and mental training, including experiences related to post-traumatic stress disorder (PTSD). These findings indicate that BDNF levels could serve as a valuable biomarker for evaluating mental health in military personnel facing operational stress, thereby offering potential insights for the prevention, diagnosis, and management of mental health disorders within this demographic (87).

A supervised exercise program in primary care has been shown to significantly improve the quality of life and emotional and social well-being of patients in advanced stages of their disease. One study identified both potential barriers and facilitators related to participation in exercise and its maintenance. However, it is crucial to promote intersectoral coordination in the socio-healthcare field to ensure integrated and continuous care for chronic patients (94).

Shuo Chan and colleagues, in 2022, investigated the effects of physical exercise on children with attention deficit hyperactivity disorder (ADHD). The researchers examined how exercise affected various aspects of ADHD, such as attention, impulsivity, and hyperactivity. The results showed that physical exercise had beneficial effects on children with ADHD. Improvements were observed in attention, concentration, and control of impulsivity after participating in regular physical exercise programs. Additionally, exercise also helped reduce symptoms of hyperactivity in these children (95).

### 6.2 Depression

More than three decades ago, physical inactivity was recognized as a significant risk factor for depression among adults. Since that time, numerous studies have reinforced the notion that exercise serves as an effective intervention for alleviating depressive symptoms. Randomized controlled trials have demonstrated that physical activity, whether performed alone or in conjunction with antidepressant medications, leads to a notable reduction in depressive symptoms compared to those observed in sedentary individuals (96). Furthermore, physical activity has been found to have an inverse relationship with anxiety sensitivity in young adults, contributing to improvements in symptoms across various anxiety disorders, including schizophrenia, autism, depression, and bipolar disorder (97). A critical element in this context is the role of psychological stress, which has been identified as a significant factor in the observed reductions in both depression and anxiety symptoms (98) Moreover, research indicates that individuals with depression often exhibit lower serum levels of brain-derived neurotrophic factor (BDNF), a relationship supported by genetic studies linking BDNF dysfunction to depressive disorders (99). Additionally, analyses have shown a marked decrease in BDNF levels within the hippocampus of patients diagnosed with major depression, further underscoring the importance of BDNF in the pathophysiology of depressive conditions (100, 101).

Conversely, antidepressant therapy has been shown to enhance BDNF expression in specific brain regions, resulting in increased levels of BDNF mRNA within just a few days of treatment. This elevation in BDNF is mediated by TrkB signaling pathways in areas such as the hippocampus and midbrain (102). Research conducted on rodent models indicates that direct administration of BDNF protein into the hippocampus may produce antidepressant-like effects (103). Furthermore, variations in BDNF function appear to affect hippocampal activity in individuals with depression, suggesting that BDNF levels may serve as potential biomarkers for the early diagnosis and timely intervention for depression and associated suicidal behaviors (104, 105).

Evidence indicates that anxiolytic medications can substantially enhance BDNF expression in the hippocampus and prefrontal cortex, resulting in increased levels of BDNF mRNA or BDNF protein (103, 106). In support of this, Jemni et al. investigated the biological and molecular mechanisms connecting various types, intensities, and durations of exercise to BDNF expression and the alleviation of depressive symptoms. Their findings highlight the potential of exercise as an effective intervention for depression (97). Additionally, Murawska et al. explored the interaction between BDNF and biological markers of depression, emphasizing the influence of physical exercise and training. Their review synthesized existing literature to elucidate how BDNF affects depression-related biomarkers, including gene expression, neuroplasticity, and overall brain function (97, 107).

### 6.3 Parkinson’s disease

Parkinson’s disease (PD) is a progressive neurological disorder characterized by the degeneration of brain cells, which negatively impacts motor control and cognitive abilities. Key pathological features of PD include the loss of dopaminergic neurons in the substantia nigra—affecting up to 70% of these cells—and the formation of Lewy bodies, which are aggregates of *α*-synuclein protein found in the remaining neurons (108). These neurobiological alterations contribute to the motor and cognitive impairments observed in PD patients (109).

Evidence indicates that exercise therapy may alleviate symptoms associated with Parkinson’s disease by enhancing neuroplasticity, improving dopaminergic function, and decreasing the accumulation of α-synuclein (110–113). Additionally, exercise therapy shows direct benefits for individuals with acquired brain injuries (114). Brain-derived neurotrophic factor (BDNF) is thought to serve as a protective agent against neurodegeneration in PD, with its levels increasing in response to exercise (115–117). This elevation in BDNF may be a key mechanism through which exercise improves clinical outcomes in PD patients. Notably, high-intensity interval training appears to have a more pronounced effect on BDNF levels than moderate-intensity continuous exercise (108, 118–120).

In animal models of Parkinson’s disease, exercise has been shown to enhance motor function by preserving nigrostriatal dopaminergic neurons, protecting mitochondrial integrity, and inhibiting the formation of Lewy bodies within the nigrostriatal pathway. These benefits contribute to the alleviation of motor symptoms, providing neuroprotection and potentially slowing disease progression through mechanisms such as improved mitochondrial function and reduced pathological accumulation of *α*-synuclein (83, 121, 122).

Studies conducted in 2016 revealed significant cognitive deficits in patients with Parkinson’s disease, evidenced by lower scores on the Repeatable Battery for the Assessment of Neuropsychological Status (RBANS) compared to healthy controls. Additionally, patients with Parkinson’s disease exhibited significantly lower serum BDNF levels (109). A positive correlation was found between BDNF levels and scores on the five RBANS indices, suggesting that diminished BDNF is associated with cognitive impairments in PD. These findings underscore the importance of BDNF in hippocampal neuroplasticity, which is critical for learning and memory, implying that reduced BDNF may contribute to the cognitive decline observed in Parkinson’s disease (9).

Despite its largely sporadic and unknown etiology, post-mortem studies have documented decreased BDNF levels in the substantia nigra compacta (SNc) of PD patients. Furthermore, evidence suggests that BDNF supports the survival of dopaminergic neurons in animal models of Parkinson’s disease. Recent studies emphasize the significance of BDNF signaling via its receptor, TrkB, in the survival of nigrostriatal dopaminergic neurons during brain aging (109). involving TrkB hypomorphic mice, which exhibit reduced receptor expression, revealed substantial loss of dopaminergic neurons in the SNc, along with diminished dopaminergic axonal terminals in the striatum and gliosis in both the SNc and striatum. Additionally, these dopaminergic neurons displayed heightened sensitivity to the neurotoxin MPTP, indicating that impaired BDNF–TrkB signaling may play a role in the progression of Parkinson’s disease (109).

Palasz et al. investigated the therapeutic potential of BDNF in the context of Parkinson’s disease, demonstrating that BDNF may offer promising therapeutic benefits by protecting dopaminergic neurons, enhancing motor function, and promoting neuronal survival in animal models of the disease. The authors also discussed potential strategies to elevate BDNF levels in the brain as a therapeutic approach for managing Parkinson’s disease (83).

### 6.4 Alzheimer’s disease

Alzheimer’s disease (AD) is a progressive and irreversible neurodegenerative disorder characterized by the degeneration and loss of neurons and synapses in the cerebral cortex and specific subcortical regions, primarily leading to symptoms such as memory loss, anxiety, and confusion (123). Research indicates that a sedentary lifestyle may contribute to an earlier onset of AD. Recent studies have shown that exercise interventions can alleviate neurodegenerative symptoms in patients with AD, and similar effects have been observed in rodent models (124). Moreover, multimodal exercise has been associated with improvements in functional capacity, suggesting that physical activity is beneficial for older adults diagnosed with Alzheimer’s disease (AD) (125).

In terms of biomarkers, aerobic training has been found to elevate plasma levels of the myokine Cathepsin B (CTSB), with changes in CTSB levels correlating with cognitive performance, while levels of BDNF have been observed to decrease. Metabolic analyses have indicated changes in polyunsaturated free fatty acids, ceramides, sphingolipids, phospholipids, and gut microbiome metabolites, alongside alterations in redox homeostasis. Notably, around 30% of the identified metabolites showed correlations with changes in BDNF levels, suggesting a possible exercise-induced metabolic regulation. The positive relationship between CTSB and cognitive performance, along with the modulation of lipid metabolites, underscores the beneficial effects of exercise on brain function, positioning CTSB as a potential marker for cognitive changes in middle-aged adults at risk for dementia (126).

In a study by Baranowski et al., both physical exercise and BDNF injections were found to positively influence APP processing enzymes and enhance cognitive function in animal models, indicating their potential as therapeutic interventions for neurodegenerative disorders like Alzheimer’s disease (127, 128).

However, discrepancies exist in the literature. For instance, Gaitán et al. explored the impact of aerobic exercise training on systemic biomarkers and cognitive function in middle-aged adults at risk for Alzheimer’s disease through a randomized controlled trial. After 26 weeks of treadmill training, an increase in the exercise-related biomarker CTSB was noted, alongside a decrease in BDNF levels. The correlation between CTSB and cognitive performance indicates a connection to brain function, with additional metabolic changes, such as increased fatty acid levels and decreased certain lipids, also observed (126).

Furthermore, Baranowski et al. investigated BDNF’s effect on the activity of the BACE1 enzyme, which is responsible for producing beta-amyloid peptides associated with Alzheimer’s disease, alongside the effects of exercise in animal models. Their findings revealed that direct BDNF treatment significantly reduced BACE1 activity in the prefrontal cortex, independent of other changes induced by exercise. Additionally, a high-fat diet was shown to lower BDNF levels in the hippocampus, while acute exercise was found to elevate BDNF levels in the prefrontal cortex (129).

### 6.5 Schizophrenia

Schizophrenia is marked by significant changes in brain morphology, including enlarged ventricles and reductions in various brain regions (130, 131). These structural alterations have been linked to positive symptoms, negative symptoms, and cognitive deficits associated with the disorder (132, 133). The activity of brain-derived neurotrophic factor (BDNF) is believed to be influenced by genetic factors, particularly the Val66Met polymorphism (134, 135). BDNF plays a vital role in neurogenesis and synaptic connectivity in the brains of individuals with schizophrenia (136). Particular attention has been directed toward the Val66Met polymorphism within the BDNF gene, which is associated with reduced brain volume, especially in areas like the hippocampus (137–139). Studies indicates that individuals carrying the Met allele tend to exhibit a smaller overall brain volume and greater reductions in specific regions compared to Val/Val homozygotes (140, 141).

Investigations into the relationship between serum BDNF levels and brain volume changes in schizophrenia have been conducted. Evidence suggests that peripheral BDNF levels are generally lower in individuals with schizophrenia compared to healthy controls (139). Nonetheless, the precise correlation between serum BDNF levels and brain volume reductions remains unclear (142–144). Further research examining the connection between BDNF and brain volume changes in schizophrenia could enhance our understanding of the disorder’s pathophysiology and potentially inform the development of new therapeutic strategies (145).

### 6.6 Multiple sclerosis (MS)

Amato and colleagues investigated (146) the effects of physical exercise, specifically lactate threshold training, in patients with multiple sclerosis (MS). In a study with eight subjects, the effects of a biweekly training program over 12 weeks were evaluated at three time points: baseline (T0), after the program (T1), and 9 months later (T2), considering physical, psychological, hematochemical parameters and dietary habits. The results showed a significant increase in brain-derived neurotrophic factor (BDNF) levels, suggesting that exercise could help improve neuronal function and reduce the progression of MS. Although dehydroepiandrosterone sulfate (DHEAS) showed a trend towards increase, it did not reach statistical significance but was associated with reduced fatigue, a common symptom in MS. Furthermore, a decrease in lactate levels was observed, indicating an improvement in lactate reutilization as an energy substrate, contributing to greater metabolic efficiency. Exercise also favored patients’ self-efficacy, improving their perception of their ability to cope with the disease’s challenges and enhancing their self-esteem. These findings support the hypothesis that physical exercise has synergistic effects on inflammation, neuroprotection, and neuronal plasticity, although further studies with larger samples and longer intervention periods are needed to confirm these results.

In addition, Brain-derived neurotrophic factor (BDNF) is a key regulator of neuroprotection, neuroplasticity, and remyelination, processes that are critically important in the context of multiple sclerosis (MS) (147). BDNF facilitates repair mechanisms within the central nervous system, which are often impaired in MS, resulting in progressive neuronal damage and increasing disability over time (147). Despite the still unresolved mechanisms underlying remyelination (148) and its variable extent among MS patients, BDNF is believed to play an essential role in these regenerative processes. Recent findings indicate that BDNF might exert beneficial effects on MS, as research showing favorable clinical improvements and MRI results predominates over studies suggesting negative effects. The role of the Val66Met polymorphism in MS remains uncertain, with studies divided on whether it represents a protective or detrimental factor (149, 150). However, the prevailing hypothesis suggests that the polymorphism may modulate BDNF secretion and exert anti-inflammatory effects, contributing to the disease’s progression in an inflammatory environment. To clarify BDNF’s impact on MS and refine therapeutic strategies, additional research involving larger patient cohorts and extended longitudinal studies is required.

## 7 Neuroprotection and exercise: therapeutic potential

In recent decades, a paradox has emerged: although life expectancy continues to rise, there has also been a concomitant increase in chronic conditions, leading to significant functional costs (151). These costs have serious implications for both health and socioeconomic factors. Cognitive decline is one condition that illustrates this issue, affecting nearly fifty million individuals globally, as reported by the World Health Organization (WHO) (152). Currently, pharmacological treatments for cognitive decline lack sufficient efficacy and often produce limited cognitive benefits (153–155). Consequently, research is increasingly focusing on non-pharmacological approaches for managing dementia and mild cognitive impairment (156). Accumulating evidence indicates that physical exercise represents the most effective non-pharmacological intervention for enhancing brain health. Studies demonstrate that exercise can diminish the risk of cognitive decline and delay the onset of dementia (157). Regular physical activity not only mitigates age-related cognitive decline but also reduces the likelihood of developing neurodegenerative diseases and psychiatric disorders (158). These protective effects are attributed to enhanced neurogenesis and neuroplasticity, which improve learning and memory. At a molecular level, brain-derived neurotrophic factor (BDNF) is integral to the neuroprotective effects of physical activity (158). Individuals who do not engage in regular exercise exhibit a significantly elevated risk of cognitive decline (159). Thus, physical exercise is proposed as an accessible, low-cost intervention with minimal side effects, allowing for benefits without requiring extensive physical activity (160, 161).

Numerous studies have shown that consistent physical activity not only enhances cardiovascular and metabolic health but also fosters neuroplasticity and neurogenesis, both of which are essential for maintaining cognitive function and preventing neuronal degeneration. The roles of physical exercise and BDNF have become central to understanding neurodegenerative disease modulation (162, 163). Furthermore, research indicates that exercise interventions positively influence BDNF levels in healthy individuals (164, 165). BDNF is crucial for neuronal survival and growth, underscoring its significance in neurological health (29). However, some researchers argue that while physical exercise promotes beneficial effects via BDNF expression, the precise cellular sources and molecular mechanisms underlying the elevated BDNF production in the brain resulting from exercise are still being elucidated (159, 161, 166, 167).

An increase in BDNF levels due to physical activity is thought to enhance adult neurogenesis and synaptogenesis, preventing neuronal loss and potentially leading to cognitive improvements and reductions in psychiatric symptoms. It is noteworthy that peripheral BDNF levels may reflect central nervous system health to some degree, with lower concentrations typically observed in individuals with psychiatric and metabolic disorders (29).

Neurodegenerative diseases are often associated with diminished BDNF levels. A recent meta-analysis examined the effects of exercise on plasma BDNF levels in individuals with various neurodegenerative disorders, including multiple sclerosis, Parkinson’s disease, mild cognitive impairment (MCI), and Alzheimer’s disease. This meta-analysis, encompassing 18 randomized controlled trials (RCTs), found that exercise interventions significantly elevated plasma BDNF levels. Notably, this increase was particularly evident in individuals with multiple sclerosis and Parkinson’s disease, while MCI showed a non-significant trend. The elevation in BDNF levels was consistent across different types of exercise, including combined, aerobic, or strength training, as well as varying weekly exercise volumes and intervention durations. The authors concluded that physical exercise interventions can enhance plasma BDNF levels in individuals with neurodegenerative disorders, indicating potential therapeutic benefits for these conditions (86).

Supporting this notion, BDNF is posited to act as a crucial mediator linking physical activity with the alleviation of neurodegenerative diseases such as Alzheimer’s and Parkinson’s disease. A recent meta-analysis by Hirsch et al. (206) reaffirmed that exercise positively impacts BDNF levels in individuals with Parkinson’s disease, although it included uncontrolled studies that may introduce bias (168). Similarly, increases in BDNF levels have been documented in patients with neurological disorders, including stroke, following aerobic exercise interventions (169).

Among the physiological mechanisms investigated, exercise is recognized for its neuroprotective role, enhancing neuronal survival, promoting angiogenesis and neurogenesis, reducing inflammation, and significantly lowering cerebrovascular risk factors (157, 168, 170–172).

In animal models, research by Nagahara et al. demonstrated that BDNF provides neuroprotective effects on neural circuits affected by Alzheimer’s disease. In transgenic mice exhibiting amyloidosis, BDNF gene administration post-disease onset reversed synaptic loss, partially normalized abnormal gene expression, enhanced cellular signaling, and restored learning and memory, independent of amyloid plaque load. Moreover, BDNF infusion in aged rats reversed cognitive decline and improved age-related gene expression disruptions. Additionally, BDNF was shown to prevent lesion-induced death of entorhinal cortical neurons in rats and adult primates, while reversing neuronal atrophy and improving cognitive decline in aged primates (173).

## 8 Discussion

Brain-derived neurotrophic factor (BDNF) plays a crucial role in neuroplasticity, neurogenesis, and neuronal protection, all of which are fundamental for maintaining optimal brain function and preventing neurodegenerative diseases. The available data suggest that physical exercise is a highly effective intervention for reducing the risk of neurological and psychological disorders, as well as preserving mood stability and neuronal integrity. Integrating exercise into treatment plans alongside conventional therapies has the potential to decrease mortality rates without introducing the side effects commonly associated with pharmacological treatments.

Neurodegenerative diseases, such as Alzheimer’s and Parkinson’s, are increasing public health challenges globally. Over 55 million individuals currently suffer from dementia, and Parkinson’s disease prevalence has doubled in recent decades, significantly impacting quality of life. Numerous scientific studies have established BDNF as a pivotal factor in promoting brain health.

One of the most promising aspects of ongoing research focuses on the regulatory effects of physical exercise on BDNF. Recent investigations have explored how various exercise types—differing in intensity and duration—modulate BDNF levels and the associated outcomes on cognition, emotional well-being, and neuroplasticity. Studies have consistently shown that high-intensity exercise induces a significant, immediate increase in BDNF levels in healthy young adults, highlighting that physical activity not only yields long-term neuroprotective effects but also induces acute changes in neurotrophic markers. Research by Dadkhah et al. (174) and Antunes et al. (175) has provided experimental and clinical evidence on the role of exercise in BDNF regulation and its positive effects on cognitive function. These findings underscore the potential of incorporating physical exercise as a complementary strategy in the prevention and treatment of neurodegenerative diseases.

Additionally, the interaction between BDNF and genetic factors, such as the BDNF Val66Met polymorphism, adds further complexity to the relationship between exercise and cognitive health. The BDNF Val66Met polymorphism has been shown to influence cognitive function, particularly when combined with regular physical exercise, suggesting that individual genetic profiles may modulate the brain’s response to exercise-induced BDNF regulation. This growing body of evidence supports the notion that physical activity can significantly impact BDNF regulation, providing protection against age-related cognitive decline and neurodegenerative disorders. However, more research is necessary to elucidate the specific mechanisms involved and optimize exercise-based interventions for diverse populations, including those with neurological conditions.

This expanding field of research holds the potential to revolutionize our understanding and management of neurodegenerative diseases, offering new strategies to enhance brain health and improve the quality of life for millions worldwide.

## References

[1] Organización Mundial de la Salud. Demencia [Internet]. Available at: https://www.who.int/es/news-room/fact-sheets/detail/dementia (Accessed March 5, 2023).

[2] Steel Z Marnane C Iranpour C Chey T Jackson JW Patel V. The global prevalence of common mental disorders: a systematic review and meta-analysis 1980-2013. Int J Epidemiol. (2014) 43:476–93. doi: 10.1093/ije/dyu038, PMID: 24648481 PMC3997379

[3] Feigin VL Nichols E Alam T Bannick MS Beghi E Blake N. Global, regional, and national burden of neurological disorders, 1990-2016: a systematic analysis for the global burden of disease study 2016. Lancet Neurol. (2019) 18:459–80. doi: 10.1016/S1474-4422(18)30499-X, PMID: 30879893 PMC6459001

[4] Organización Mundial de la Salud. Enfermedad de Parkinson [Internet]. Available at: https://www.who.int/es/news-room/fact-sheets/detail/parkinson-disease (Accessed August 9, 2023).

[5] Tsai SJ. Critical issues in BDNF Val 66Met genetic studies of neuropsychiatric disorders. Front Mol Neurosci. (2018) 11:156. doi: 10.3389/fnmol.2018.00156, PMID: 29867348 PMC5962780

[6] Lima Giacobbo B Doorduin J Klein HC Dierckx RAJO Bromberg E de Vries EFJ. Brain-derived Neurotrophic factor in brain disorders: focus on neuroinflammation. Mol Neurobiol. (2019) 56:3295–312. doi: 10.1007/s12035-018-1283-6, PMID: 30117106 PMC6476855

[7] Ng TKS Ho CSH Tam WWS Kua EH Ho RCM. Decreased serum brain-derived Neurotrophic factor (BDNF) levels in patients with Alzheimer’s disease (AD): a systematic review and meta-analysis. Int J Mol Sci. (2019) 20:257. doi: 10.3390/ijms20020257, PMID: 30634650 PMC6358753

[8] Janakiraman U Manivasagam T Justin Thenmozhi A Dhanalakshmi C Essa MM Song BJ. Chronic mild stress augments MPTP induced neurotoxicity in a murine model of Parkinson’s disease. Physiol Behav. (2017) 173:132–43. doi: 10.1016/j.physbeh.2017.01.046, PMID: 28185878

[9] Wang Y Liu H Zhang BS Soares JC Zhang XY. Low BDNF is associated with cognitive impairments in patients with Parkinson’s disease. Parkinsonism Relat Disord. (2016) 29:66–71. doi: 10.1016/j.parkreldis.2016.05.023, PMID: 27245919

[10] Azoulay D Urshansky N Karni A. Low and dysregulated BDNF secretion from immune cells of MS patients is related to reduced neuroprotection. J Neuroimmunol. (2008) 195:186–93. doi: 10.1016/j.jneuroim.2008.01.010, PMID: 18329726

[11] Patanella AK Zinno M Quaranta D Nociti V Frisullo G Gainotti G. Correlations between peripheral blood mononuclear cell production of BDNF, TNF-alpha, IL-6, IL-10 and cognitive performances in multiple sclerosis patients. J Neurosci Res. (2010) 88:1106–12. doi: 10.1002/jnr.22276, PMID: 19885866

[12] Pradhan J Noakes PG Bellingham MC. The role of altered BDNF/Trk B Signaling in amyotrophic lateral sclerosis. Front Cell Neurosci. (2019) 13:13. doi: 10.3389/fncel.2019.00368, PMID: 31456666 PMC6700252

[13] Almeida RD Manadas BJ Melo CV Gomes JR Mendes CS Grãos MM. Neuroprotection by BDNF against glutamate-induced apoptotic cell death is mediated by ERK and PI3-kinase pathways. Cell Death Differ. (2005) 12:1329–43. doi: 10.1038/sj.cdd.4401662, PMID: 15905876

[14] Miranda M Morici JF Zanoni MB Bekinschtein P. Brain-derived Neurotrophic factor: a key molecule for memory in the healthy and the pathological brain. Front Cell Neurosci. (2019) 13:13. doi: 10.3389/fncel.2019.00363, PMID: 31440144 PMC6692714

[15] Fernández-Rodríguez R Álvarez-Bueno C Martínez-Ortega IA Martínez-Vizcaíno V Mesas AE Notario-Pacheco B. Immediate effect of high-intensity exercise on brain-derived neurotrophic factor in healthy young adults: a systematic review and meta-analysis. J Sport Health Sci. (2022) 11:367–75. doi: 10.1016/j.jshs.2021.08.004, PMID: 34481089 PMC9189701

[16] Edman S Horwath O Van der Stede T Blackwood SJ Moberg I Strömlind H. Pro-brain-derived Neurotrophic factor (BDNF), but not mature BDNF, is expressed in human skeletal muscle: implications for exercise-induced neuroplasticity. Function. (2024) 5:zqae005. doi: 10.1093/function/zqae005, PMID: 38706964 PMC11065112

[17] Ernfors P Ibáñez CF Ebendal T Olson L Persson H. Molecular cloning and neurotrophic activities of a protein with structural similarities to nerve growth factor: developmental and topographical expression in the brain. Proc Natl Acad Sci USA. (1990) 87:5454–8. doi: 10.1073/pnas.87.14.5454, PMID: 2164684 PMC54343

[18] Lessmann V Gottmann K Malcangio M. Neurotrophin secretion: current facts and future prospects. Prog Neurobiol. (2003) 69:341–74. doi: 10.1016/S0301-0082(03)00019-4, PMID: 12787574

[19] Donovan MJ Miranda RC Kraemer R McCaffrey TA Tessarollo L Mahadeo D. Neurotrophin and neurotrophin receptors in vascular smooth muscle cells: regulation of expression in response to injury. Am J Pathol. (1995) 147:309–24. PMID: 7639328 PMC1869811

[20] Lommatzsch M Zingler D Schuhbaeck K Schloetcke K Zingler C Schuff-Werner P. The impact of age, weight and gender on BDNF levels in human platelets and plasma. Neurobiol Aging. (2005) 26:115–23. doi: 10.1016/j.neurobiolaging.2004.03.002, PMID: 15585351

[21] Tamura S Suzuki H Hirowatari Y Hatase M Nagasawa A Matsuno K. Release reaction of brain-derived neurotrophic factor (BDNF) through PAR1 activation and its two distinct pools in human platelets. Thromb Res. (2011) 128:e55–61. doi: 10.1016/j.thromres.2011.06.002, PMID: 21924459

[22] Nakahashi T Fujimura H Altar CA Li J Kambayashi JI Tandon NN. Vascular endothelial cells synthesize and secrete brain-derived neurotrophic factor. FEBS Lett. (2000) 470:113–7. doi: 10.1016/S0014-5793(00)01302-8, PMID: 10734218

[23] Tang SW Chu E Hui T Helmeste D Law C. Influence of exercise on serum brain-derived neurotrophic factor concentrations in healthy human subjects. Neurosci Lett. (2008) 431:62–5. doi: 10.1016/j.neulet.2007.11.019, PMID: 18068900

[24] Erickson KI Miller DL Roecklein KA. The aging hippocampus: interactions between exercise, depression, and BDNF. Neuroscientist. (2012) 18:82–97. doi: 10.1177/1073858410397054, PMID: 21531985 PMC3575139

[25] de Azevedo KPM de Oliveira VH de Medeiros GCBS ÁNS M García DÁ Martínez DG. The effects of exercise on BDNF levels in adolescents: a systematic review with meta-analysis. Int J Environ Res Public Health. (2020) 17:1–14. doi: 10.3390/ijerph17176056, PMID: 32825341 PMC7503913

[26] Erickson KI Voss MW Prakash RS Basak C Szabo A Chaddock L. Exercise training increases size of hippocampus and improves memory. Proc Natl Acad Sci USA. (2011) 108:3017–22. doi: 10.1073/pnas.1015950108, PMID: 21282661 PMC3041121

[27] Cefis M Prigent-Tessier A Quirié A Pernet N Marie C Garnier P. The effect of exercise on memory and BDNF signaling is dependent on intensity. Brain Struct Funct. (2019) 224:1975–85. doi: 10.1007/s00429-019-01889-7, PMID: 31089854

[28] Müller P Duderstadt Y Lessmann V Müller NG. Lactate and BDNF: key mediators of exercise induced neuroplasticity? J Clin Med. (2020) 9:1136. doi: 10.3390/jcm9041136, PMID: 32326586 PMC7230639

[29] Dinoff A Herrmann N Swardfager W Liu CS Sherman C Chan S. The effect of exercise training on resting concentrations of peripheral brain-derived Neurotrophic factor (BDNF): a meta-analysis. PLoS One. (2016) 11:e0163037. doi: 10.1371/journal.pone.0163037, PMID: 27658238 PMC5033477

[30] Numakawa T Suzuki S Kumamaru E Adachi N Richards M Kunugi H. BDNF function and intracellular signaling in neurons. Histol Histopathol. (2010) 25:237–58. doi: 10.14670/HH-25.237, PMID: 20017110

[31] Anand K Dhikav V. Hippocampus in health and disease: An overview. Ann Indian Acad Neurol. (2012) 15:239–46. doi: 10.4103/0972-2327.104323, PMID: 23349586 PMC3548359

[32] Howells DW Porritt MJ Wong JYF Batchelor PE Kalnins R Hughes AJ. Reduced BDNF mRNA expression in the Parkinson’s disease substantia nigra. Exp Neurol. (2000) 166:127–35. doi: 10.1006/exnr.2000.7483, PMID: 11031089

[33] Michalski B Fahnestock M. Pro-brain-derived neurotrophic factor is decreased in parietal cortex in Alzheimer’s disease. Mol Brain Res. (2003) 111:148–54. doi: 10.1016/S0169-328X(03)00003-2, PMID: 12654514

[34] Zhao WQ Chen H Quon MJ Alkon DL. Insulin and the insulin receptor in experimental models of learning and memory. Eur J Pharmacol. (2004) 490:71–81. doi: 10.1016/j.ejphar.2004.02.045, PMID: 15094074

[35] Egan MF Kojima M Callicott JH Goldberg TE Kolachana BS Bertolino A. The BDNF val 66met polymorphism affects activity-dependent secretion of BDNF and human memory and hippocampal function. Cell. (2003) 112:257–69. doi: 10.1016/S0092-8674(03)00035-7, PMID: 12553913

[36] Ieraci A Madaio AI Mallei A Lee FS Popoli M. Brain-derived Neurotrophic factor Val 66Met human polymorphism impairs the beneficial exercise-induced neurobiological changes in mice. Neuropsychopharmacology. (2016) 41:3070–9. doi: 10.1038/npp.2016.120, PMID: 27388329 PMC5101555

[37] Hopkins ME Davis FC Van Tieghem MR Whalen PJ Bucci DJ. Differential effects of acute and regular physical exercise on cognition and affect. Neuroscience. (2012) 215:59–68. doi: 10.1016/j.neuroscience.2012.04.056, PMID: 22554780 PMC3374855

[38] Li S Weinstein G Zare H Teumer A Völker U Friedrich N. The genetics of circulating BDNF: towards understanding the role of BDNF in brain structure and function in middle and old ages. Brain Commun. (2020) 2:fcaa176. doi: 10.1093/braincomms/fcaa176, PMID: 33345186 PMC7734441

[39] Terracciano A Piras MG Lobina M Mulas A Meirelles O Sutin AR. Genetics of serum BDNF: meta-analysis of the Val 66Met and genome-wide association study. World J Biol Psychiatry. (2013) 14:583–9. doi: 10.3109/15622975.2011.616533, PMID: 22047184 PMC3288597

[40] Pedersen BK Saltin B. Exercise as medicine - evidence for prescribing exercise as therapy in 26 different chronic diseases. Scand J Med Sci Sports. (2015) 25:1–72. doi: 10.1111/sms.12581, PMID: 26606383

[41] Fiuza-Luces C Garatachea N Berger NA Lucia A. Exercise is the real polypill. Physiology (Bethesda). (2013) 28:330–58. doi: 10.1152/physiol.00019.2013, PMID: 23997192

[42] Hoffmann TC Maher CG Briffa T Sherrington C Bennell K Alison J. Prescribing exercise interventions for patients with chronic conditions. CMAJ. (2016) 188:510–8. doi: 10.1503/cmaj.150684, PMID: 26976965 PMC4835280

[43] Pollán M Casla-Barrio S Alfaro J Esteban C Segui-Palmer MA Lucia A. Exercise and cancer: a position statement from the Spanish Society of Medical Oncology. Clin Transl Oncol. (2020) 22:1710–29. doi: 10.1007/s12094-020-02312-y, PMID: 32052383 PMC7423809

[44] Lammers MD Aneli NM de Oliveira GG de Oliveira Maciel SFV Zanini D Manica A. The anti-inflammatory effect of resistance training in hypertensive women: the role of purinergic signaling. J Hypertens. (2020) 38:2490–500. doi: 10.1097/HJH.0000000000002578, PMID: 32694341

[45] Liguori. Manual ACSM para la valoración y prescripción, 4ed [Internet]. Available at: https://user-biackli. cld.bz/Liguori-Manual-ACSM-para-la-valoracion-y-prescripcion-4ed/4/

[46] Vivar C Peterson BD van Praag H. Running rewires the neuronal network of adult-born dentate granule cells. NeuroImage. (2016) 131:29–41. doi: 10.1016/j.neuroimage.2015.11.031, PMID: 26589333 PMC6003242

[47] Van Der Borght K Kóbor-Nyakas DÉ Klauke K Eggen BJL Nyakas C Van Der Zee EA. Physical exercise leads to rapid adaptations in hippocampal vasculature: temporal dynamics and relationship to cell proliferation and neurogenesis. Hippocampus. (2009) 19:928–36. doi: 10.1002/hipo.20545, PMID: 19212941

[48] Gruart A Sciarretta C Valenzuela-Harrington M Delgado-García JM Minichiello L. Mutation at the Trk B PLC {gamma}-docking site affects hippocampal LTP and associative learning in conscious mice. Learn Mem. (2007) 14:54–62. doi: 10.1101/lm.428307, PMID: 17272652 PMC1838546

[49] Yu Q Li X Wang J Li Y. Effect of exercise training on long-term potentiation and NMDA receptor channels in rats with cerebral infarction. Exp Ther Med. (2013) 6:1431–6. doi: 10.3892/etm.2013.1319, PMID: 24250723 PMC3829711

[50] Sáenz JC. Beneficios del Ejercicio Físico sobre la Neuroplasticidad y la Cognición. Neuro Rehab News. (2021) Octubre. doi: 10.37382/nrn.Octubre.2017.524

[51] Farmer J Zhao X Van Praag H Wodtke K Gage FH Christie BR. Effects of voluntary exercise on synaptic plasticity and gene expression in the dentate gyrus of adult male Sprague–Dawley rats in vivo. Neuroscience. (2004) 124:71–9. doi: 10.1016/j.neuroscience.2003.09.029, PMID: 14960340

[52] Notaras M van den Buuse M. Brain-derived Neurotrophic factor (BDNF): novel insights into regulation and genetic variation. Neuroscientist. (2019) 25:434–54. doi: 10.1177/1073858418810142, PMID: 30387693

[53] Ferris LT Williams JS Shen CL. The effect of acute exercise on serum brain-derived neurotrophic factor levels and cognitive function. Med Sci Sports Exerc. (2007) 39:728–34. doi: 10.1249/mss.0b013e31802f04c7, PMID: 17414812

[54] Griffin ÉW Mullally S Foley C Warmington SA O’Mara SM Kelly ÁM. Aerobic exercise improves hippocampal function and increases BDNF in the serum of young adult males. Physiol Behav. (2011) 104:934–41. doi: 10.1016/j.physbeh.2011.06.005, PMID: 21722657

[55] Cunha C Brambilla R Thomas KL. A simple role for BDNF in learning and memory? Front Mol Neurosci. (2010) 3:865. doi: 10.3389/neuro.02.001.2010, PMID: 20162032 PMC2821174

[56] Montero-Herrera B Jaime Fornaguera-Trías A Montero-Herrera PB. Ejercicio y algunos mecanismos moleculares que subyacen a una mejora del desempeño en tareas cognitivas. Rev. Ib. CC. Act. Fis. Dep. (2020) 9:75–94. doi: 10.24310/riccafd.2020.v9i1.8303

[57] Voss MW Heo S Prakash RS Erickson KI Alves H Chaddock L. The influence of aerobic fitness on cerebral white matter integrity and cognitive function in older adults: results of a one-year exercise intervention. Hum Brain Mapp. (2013) 34:2972–85. doi: 10.1002/hbm.22119, PMID: 22674729 PMC4096122

[58] Duzel E Van Praag H Sendtner M. Can physical exercise in old age improve memory and hippocampal function? Brain. (2016) 139:662–73. doi: 10.1093/brain/awv407, PMID: 26912638 PMC4766381

[59] Kramer AF Colcombe S. Fitness effects on the cognitive function of older adults: a meta-analytic study—revisited. Perspect Psychol Sci. (2018) 13:213–7. doi: 10.1177/1745691617707316, PMID: 29592650

[60] Åberg MAI Pedersen NL Torén K Svartengren M Bäckstrand B Johnsson T. Cardiovascular fitness is associated with cognition in young adulthood. Proc Natl Acad Sci USA. (2009) 106:20906–11. doi: 10.1073/pnas.0905307106, PMID: 19948959 PMC2785721

[61] Radka SF Hoist PA Fritsche M Altar CA. Presence of brain-derived neurotrophic factor in brain and human and rat but not mouse serum detected by a sensitive and specific immunoassay. Brain Res. (1996) 709:122–30. doi: 10.1016/0006-8993(95)01321-0, PMID: 8869564

[62] Yoshimura R Sugita-Ikenouchi A Hori H Umene-Nakano W Hayashi K Katsuki A. A close correlation between plasma and serum levels of brain-derived neurotrophic factor (BDNF) in healthy volunteers. Int J Psychiatry Clin Pract. (2010) 14:220–2. doi: 10.3109/13651501003748560, PMID: 24917323

[63] Silva ND Vicente PB Valdivia PM. Factor neurotrófico derivado del cerebro como marcador de conducta suicida en pacientes con trastorno depresivo mayor. Rev Chil Neuropsiquiatr. (2015) 53:44–52. doi: 10.4067/S0717-92272015000100006

[64] Tarassova O Ekblom MM Moberg M Lövdén M Nilsson J. Peripheral BDNF response to physical and cognitive exercise and its association with cardiorespiratory fitness in healthy older adults. Front Physiol. (2020) 11:1080. doi: 10.3389/fphys.2020.01080, PMID: 32982796 PMC7477111

[65] El Hayek L Khalifeh M Zibara V Abi Assaad R Emmanuel N Karnib N. Lactate mediates the effects of exercise on learning and memory through SIRT1-dependent activation of hippocampal brain-derived Neurotrophic factor (BDNF). J Neurosci. (2019) 39:2369–82. doi: 10.1523/JNEUROSCI.1661-18.2019, PMID: 30692222 PMC6435829

[66] Stimpson NJ Davison G Javadi AH. Joggin’ the noggin: towards a physiological understanding of exercise-induced cognitive benefits. Neurosci Biobehav Rev. (2018) 88:177–86. doi: 10.1016/j.neubiorev.2018.03.018, PMID: 29572187

[67] Alomari MA Khabour OF Alzoubi KH Alzubi MA. Forced and voluntary exercises equally improve spatial learning and memory and hippocampal BDNF levels. Behav Brain Res. (2013) 247:34–9. doi: 10.1016/j.bbr.2013.03.007, PMID: 23499703

[68] Maass A Düzel S Brigadski T Goerke M Becke A Sobieray U. Relationships of peripheral IGF-1, VEGF and BDNF levels to exercise-related changes in memory, hippocampal perfusion and volumes in older adults. NeuroImage. (2016) 131:142–54. doi: 10.1016/j.neuroimage.2015.10.084, PMID: 26545456

[69] Piepmeier AT Etnier JL Wideman L Berry NT Kincaid Z Weaver MA. A preliminary investigation of acute exercise intensity on memory and BDNF isoform concentrations. Eur J Sport Sci. (2020) 20:819–30. doi: 10.1080/17461391.2019.1660726, PMID: 31495276

[70] Neeper SA Gómez-Pinilla F Choi J Cotman CW. Physical activity increases mRNA for brain-derived neurotrophic factor and nerve growth factor in rat brain. Brain Res. (1996) 726:49–56. doi: 10.1016/0006-8993(96)00273-9, PMID: 8836544

[71] Wan J Ma L Jiao X Dong W Lin J Qiu Y. Impaired synaptic plasticity and decreased excitability of hippocampal glutamatergic neurons mediated by BDNF downregulation contribute to cognitive dysfunction in mice induced by repeated neonatal exposure to ketamine. CNS Neurosci Ther. (2024) 30:e14604. doi: 10.1111/cns.14604, PMID: 38332635 PMC10853651

[72] Hao L Yang Z Gong P Lei J. Maintenance of postsynaptic neuronal excitability by a positive feedback loop of postsynaptic BDNF expression. Cogn Neurodyn. (2018) 12:403–16. doi: 10.1007/s11571-018-9479-z, PMID: 30137877 PMC6048012

[73] Jiang X Tian F Du Y Copeland NG Jenkins NA Tessarollo L. BHLHB2 controls *Bdnf* promoter 4 activity and neuronal excitability. J Neurosci. (2008) 28:1118–30. doi: 10.1523/JNEUROSCI.2262-07.2008, PMID: 18234890 PMC6671398

[74] Jun R Zhang W Beacher NJ Zhang Y Li Y Lin DT. Dysbindin-1, BDNF, and GABAergic transmission in schizophrenia. Front Psychiatry. (2022) 13:13. doi: 10.3389/fpsyt.2022.876749, PMID: 35815020 PMC9258742

[75] Xu L Zhu L Zhu L Chen D Cai K Liu Z. Moderate exercise combined with enriched environment enhances learning and memory through BDNF/Trk B Signaling pathway in rats. Int J Environ Res Public Health. (2021) 18:8283. doi: 10.3390/ijerph18168283, PMID: 34444034 PMC8392212

[76] Tyler WJ Pozzo-Miller LD. BDNF enhances quantal neurotransmitter release and increases the number of docked vesicles at the active zones of hippocampal excitatory synapses. J Neurosci. (2001) 21:4249–58. doi: 10.1523/JNEUROSCI.21-12-04249.2001, PMID: 11404410 PMC2806848

[77] Ding Q Vaynman S Akhavan M Ying Z Gomez-Pinilla F. Insulin-like growth factor I interfaces with brain-derived neurotrophic factor-mediated synaptic plasticity to modulate aspects of exercise-induced cognitive function. Neuroscience. (2006) 140:823–33. doi: 10.1016/j.neuroscience.2006.02.084, PMID: 16650607

[78] Haapasalo A Sipola I Larsson K Åkerman KEO Stoilov P Stamm S. Regulation of TRKB surface expression by brain-derived neurotrophic factor and truncated TRKB isoforms. J Biol Chem. (2002) 277:43160–7. doi: 10.1074/jbc.M205202200, PMID: 12202482

[79] Ballesteros Carrasco JJ. El papel del sistema BDNF-Trk B en la plasticidad sináptica independiente de receptores de tipo NMDA inducida por xantinas. El papel del sistema BDNF-Trk B en la plasticidad sináptica independiente de receptores de tipo NMDA inducida por xantinas. (2015)

[80] Galeano HP. El papel del ejercicio físico en la inducción de BDNF y sus vías de señalización en el sistema nervioso central. Aplicación neurobiológica en modelos sanos y terapéutica en la enfermedad de Alzheimer. (2014).

[81] Koshimizu H Kiyosue K Hara T Hazama S Suzuki S Uegaki K. Multiple functions of precursor BDNF to CNS neurons: negative regulation of neurite growth, spine formation and cell survival. Mol Brain. (2009) 2:1–19. doi: 10.1186/1756-6606-2-27, PMID: 19674479 PMC2743674

[82] Castrén E Kojima M. Brain-derived neurotrophic factor in mood disorders and antidepressant treatments. Neurobiol Dis. (2017) 97:119–26. doi: 10.1016/j.nbd.2016.07.010, PMID: 27425886

[83] Palasz E Wysocka A Gasiorowska A Chalimoniuk M Niewiadomski W Niewiadomska G. BDNF as a promising therapeutic agent in Parkinson’s disease. Int J Mol Sci. (2020) 21:1–23. doi: 10.3390/ijms21031170, PMID: 32050617 PMC7037114

[84] Lu B Nagappan G Guan X Nathan PJ Wren P. BDNF-based synaptic repair as a disease-modifying strategy for neurodegenerative diseases. Nat Rev Neurosci. (2013) 14:401–16. doi: 10.1038/nrn3505, PMID: 23674053

[85] Lu B Nagappan G Lu Y. BDNF and synaptic plasticity, cognitive function, and dysfunction. Handb Exp Pharmacol. (2015) 220:223–50. doi: 10.1007/978-3-642-45106-5_9, PMID: 24668475

[86] Ruiz-González D Hernández-Martínez A Valenzuela PL Morales JS Soriano-Maldonado A. Effects of physical exercise on plasma brain-derived neurotrophic factor in neurodegenerative disorders: a systematic review and meta-analysis of randomized controlled trials. Neurosci Biobehav Rev. (2021) 128:394–405. doi: 10.1016/j.neubiorev.2021.05.025, PMID: 34087277

[87] Martínez-Salazar IN Martínez-Cuazitl A Comoto-Santacruz DA Ríos-Vela D Díaz-Sánchez Y Chavez-Velasco AS. Changes in brain-derived neurotrophic factor by different kind of military training. Rev Sanid Milit. (2022) 76:1–11. doi: 10.56443/rsm.v76i4.320

[88] Campbell TS Donoghue KM Ghosh U Nelson CM Roth TL. Early life stress affects Bdnf regulation: a role for exercise interventions. Int J Mol Sci. (2022) 23:1–14. doi: 10.3390/ijms231911729, PMID: 36233029 PMC9569911

[89] Booij L Richard T Szyf M Benkelfat C. Genetic and early environmental influences on the serotonin system: consequences for brain development and risk for psychopathology. J Psychiatry Neurosci. (2015) 40:5–18. doi: 10.1503/jpn.140099, PMID: 25285876 PMC4275332

[90] Misiak B Stramecki F Gawęda Ł Prochwicz K Sąsiadek MM Moustafa AA. Interactions between variation in candidate genes and environmental factors in the Etiology of schizophrenia and bipolar disorder: a systematic review. Mol Neurobiol. (2018) 55:5075–100. doi: 10.1007/s12035-017-0708-y, PMID: 28822116 PMC5948257

[91] Xie B Liu Z Liu W Jiang L Zhang R Cui D. DNA methylation and tag SNPs of the BDNF gene in conversion of amnestic mild cognitive impairment into Alzheimer’s disease: a cross-sectional cohort study. J Alzheimers Dis. (2017) 58:263–74. doi: 10.3233/JAD-170007, PMID: 28387675

[92] Chagnon YC Potvin O Hudon C Préville M. DNA methylation and single nucleotide variants in the brain-derived neurotrophic factor (BDNF) and oxytocin receptor (OXTR) genes are associated with anxiety/depression in older women. Front Genet. (2015) 6:230. doi: 10.3389/fgene.2015.00230, PMID: 26175754 PMC4485183

[93] Zhao M Chen L Yang J Han D Fang D Qiu X. BDNF Val 66Met polymorphism, life stress and depression: a meta-analysis of gene-environment interaction. J Affect Disord. (2018) 227:226–35. doi: 10.1016/j.jad.2017.10.024, PMID: 29102837

[94] Arietaleanizbeaskoa MS Sancho A Olazabal I Moreno C Gil E Garcia-Alvarez A. Effectiveness of physical exercise for people with chronic diseases: the EFIKRONIK study protocol for a hybrid, clinical and implementation randomized trial. BMC Fam Pract. (2020) 21:227. doi: 10.1186/s12875-020-01298-4, PMID: 33158422 PMC7648284

[95] Chan YS Jang JT Ho CS. Effects of physical exercise on children with attention deficit hyperactivity disorder. Biom J. (2022) 45:265–70. doi: 10.1016/j.bj.2021.11.011, PMID: 34856393 PMC9250090

[96] Puccinelli PJ da Costa TS Seffrin A de Lira CAB Vancini RL Nikolaidis PT. Reduced level of physical activity during COVID-19 pandemic is associated with depression and anxiety levels: an internet-based survey. BMC Public Health. (2021) 21:1–11. doi: 10.1186/s12889-021-10470-z 33648487 PMC7919983

[97] Jemni M Zaman R Carrick FR Clarke ND Marina M Bottoms L. Exercise improves depression through positive modulation of brain-derived neurotrophic factor (BDNF). A review based on 100 manuscripts over 20 years. Front Physiol. (2023) 14:14. doi: 10.3389/fphys.2023.1102526, PMID: 36969600 PMC10030936

[98] Lee S Doulames V Donnelly M Levasseaur J Shea TB. Environmental enrichment can prevent cognitive decline induced by dietary oxidative challenge. J Alzheimers Dis. (2012) 28:497–501. doi: 10.3233/JAD-2011-111562, PMID: 22045493

[99] Deveci A Aydemir O Taskin O Taneli F Esen-Danaci A. Serum BDNF levels in suicide attempters related to psychosocial stressors: a comparative study with depression. Neuropsychobiology. (2007) 56:93–7. doi: 10.1159/000111539, PMID: 18037819

[100] Duman RS. Neuronal damage and protection in the pathophysiology and treatment of psychiatric illness: stress and depression. Dialogues Clin Neurosci. (2009) 11:239–55. doi: 10.31887/DCNS.2009.11.3/rsduman, PMID: 19877493 PMC3181922

[101] Kishi T Yoshimura R Ikuta T Iwata N. Brain-derived Neurotrophic factor and major depressive disorder: evidence from meta-analyses. Front Psychiatry. (2018) 8:308. doi: 10.3389/fpsyt.2017.00308 29387021 PMC5776079

[102] Heise C Gardoni F Culotta L di Luca M Verpelli C Sala C. Elongation factor-2 phosphorylation in dendrites and the regulation of dendritic mRNA translation in neurons. Front Cell Neurosci. (2014) 8:8(FEB). doi: 10.3389/fncel.2014.00035, PMID: 24574971 PMC3918593

[103] Notaras M van den Buuse M. Neurobiology of BDNF in fear memory, sensitivity to stress, and stress-related disorders. Mol Psychiatry. (2020) 25:2251–74. doi: 10.1038/s41380-019-0639-2, PMID: 31900428

[104] Polyakova M Stuke K Schuemberg K Mueller K Schoenknecht P Schroeter ML. BDNF as a biomarker for successful treatment of mood disorders: a systematic & quantitative meta-analysis. J Affect Disord. (2015) 174:432–40. doi: 10.1016/j.jad.2014.11.044, PMID: 25553404

[105] Li W Ali T Zheng C He K Liu Z Shah FA. Anti-depressive-like behaviors of APN KO mice involve Trkb/BDNF signaling related neuroinflammatory changes. Mol Psychiatry. (2022) 27:1047–58. doi: 10.1038/s41380-021-01327-3, PMID: 34642455

[106] Li YJ Xu M Gao ZH Wang YQ Yue Z Zhang YX. Alterations of serum levels of BDNF-related mi RNAs in patients with depression. PLoS One. (2013) 8:1–7. doi: 10.1371/journal.pone.0063648, PMID: 23704927 PMC3660391

[107] Murawska-Ciałowicz E Wiatr M Ciałowicz M de Assis GG Borowicz W Rocha-Rodrigues S. BDNF impact on biological markers of depression—role of physical exercise and training. Int J Environ Res Public Health. (2021) 18:1–21. doi: 10.3390/ijerph18147553, PMID: 34300001 PMC8307197

[108] Baquet ZC Bickford PC Jones KR. Brain-derived neurotrophic factor is required for the establishment of the proper number of dopaminergic neurons in the substantia nigra pars compacta. J Neurosci. (2005) 25:6251–9. doi: 10.1523/JNEUROSCI.4601-04.2005, PMID: 15987955 PMC6725062

[109] Baydyuk M Nguyen MT Xu B. Chronic deprivation of Trk B signaling leads to selective late-onset nigrostriatal dopaminergic degeneration. Exp Neurol. (2011) 228:118–25. doi: 10.1016/j.expneurol.2010.12.018, PMID: 21192928 PMC3041148

[110] Aron L Klein R. Repairing the parkinsonian brain with neurotrophic factors. Trends Neurosci. (2011) 34:88–100. doi: 10.1016/j.tins.2010.11.001, PMID: 21144600

[111] Lin LFH Doherty DH Lile JD Bektesh S Collins F. GDNF: a glial cell line-derived neurotrophic factor for midbrain dopaminergic neurons. Science. (1993) 260:1130–2. doi: 10.1126/science.8493557, PMID: 8493557

[112] Hyman C Hofer M Barde YA Juhasz M Yancopoulos GD Squinto SP. BDNF is a neurotrophic factor for dopaminergic neurons of the substantia nigra. Nature. (1991) 350:230–2. doi: 10.1038/350230a0, PMID: 2005978

[113] Tome D Fonseca C Campos F Baltazar G. Role of neurotrophic factors in Parkinson’s disease. Curr Pharm Des. (2017) 23:809–38. doi: 10.2174/1381612822666161208120422, PMID: 27928963

[114] Rotondo R Proietti S Perluigi M Padua E Stocchi F Fini M. Physical activity and neurotrophic factors as potential drivers of neuroplasticity in Parkinson’s disease: a systematic review and meta-analysis. Ageing Res Rev. (2023) 92:102089. doi: 10.1016/j.arr.2023.102089, PMID: 37844764

[115] Pałasz E Bąk A Gąsiorowska A Niewiadomska G. The role of trophic factors and inflammatory processes in physical activity-induced neuroprotection in Parkinson’s disease. Postepy Hig Med Dosw (Online). (2017) 71:713–26. doi: 10.5604/01.3001.0010.3850, PMID: 28894046

[116] Ziebell M Khalid U Klein AB Aznar S Thomsen G Jensen P. Striatal dopamine transporter binding correlates with serum BDNF levels in patients with striatal dopaminergic neurodegeneration. Neurobiol Aging. (2012) 33:428.e1–5. doi: 10.1016/j.neurobiolaging.2010.11.010, PMID: 21176997

[117] Tajiri N Yasuhara T Shingo T Kondo A Yuan W Kadota T. Exercise exerts neuroprotective effects on Parkinson’s disease model of rats. Brain Res. (2010) 1310:200–7. doi: 10.1016/j.brainres.2009.10.075, PMID: 19900418

[118] O’Callaghan A Harvey M Houghton D Gray WK Weston KL Oates LL. Comparing the influence of exercise intensity on brain-derived neurotrophic factor serum levels in people with Parkinson’s disease: a pilot study. Aging Clin Exp Res. (2020) 32:1731–8. doi: 10.1007/s40520-019-01353-w, PMID: 31606860

[119] Fiorelli CM Ciolac EG Simieli L Silva FA Fernandes B Christofoletti G. Differential acute effect of high-intensity interval or continuous moderate exercise on cognition in individuals with Parkinson’s disease. J Phys Act Health. (2019) 16:157–64. doi: 10.1123/jpah.2018-0189, PMID: 30626260

[120] Fernandes B Barbieri FA Arthuso FZ Silva FA Moretto GF Imaizumi LFI. High-intensity interval versus moderate-intensity continuous training in individuals with Parkinson’s disease: hemodynamic and functional adaptation. J Phys Act Health. (2020) 17:85–91. doi: 10.1123/jpah.2018-0588, PMID: 31810064

[121] Drinkut A Tillack K Meka DP Schulz JB Kügler S Kramer ER. Ret is essential to mediate GDNF’s neuroprotective and neuroregenerative effect in a Parkinson disease mouse model. Cell Death Dis. (2016) 7:e2359–e 2359. doi: 10.1038/cddis.2016.263, PMID: 27607574 PMC5059866

[122] Zhao L He LX Huang SN Gong LJ Li L Lv YY. Protection of dopamine neurons by vibration training and up-regulation of brain-derived neurotrophic factor in a MPTP mouse model of Parkinson’s disease. Physiol Res. (2014) 63:649–57. doi: 10.33549/physiolres.932743, PMID: 24908088

[123] Janeiro MH Ardanaz CG Sola-Sevilla N Dong J Cortés-Erice M Solas M. Biomarcadores en la enfermedad de Alzheimer. Advances in Laboratory Medicine. (2021) 2:39–50. doi: 10.1515/almed-2020-0109, PMID: 37359199 PMC10197496

[124] Rolland Y Pillard F Klapouszczak A Reynish E Thomas D Andrieu S. Exercise program for nursing home residents with Alzheimer’s disease: a 1-year randomized, controlled trial. J Am Geriatr Soc. (2007) 55:158–65. doi: 10.1111/j.1532-5415.2007.01035.x, PMID: 17302650

[125] Braz de Oliveira MP Moreira Padovez RD Serrão PR de Noronha MA Cezar NO Andrade LP. Effectiveness of physical exercise at improving functional capacity in older adults living with Alzheimer’s disease: a systematic review of randomized controlled trials. Disabil Rehabil. (2023) 45:391–402. doi: 10.1080/09638288.2022.2037744, PMID: 35171074

[126] Gaitán JM Moon HY Stremlau M Dubal DB Cook DB Okonkwo OC. Effects of aerobic exercise training on systemic biomarkers and cognition in late middle-aged adults at risk for Alzheimer’s disease. Front Endocrinol (Lausanne). (2021) 12:12. doi: 10.3389/fendo.2021.660181, PMID: 34093436 PMC8173166

[127] Baranowski BJ Mohammad A Finch MS Brown A Dhaliwal R Marko DM. Exercise training and BDNF injections alter amyloid precursor protein (APP) processing enzymes and improve cognition. J Appl Physiol (1985). (2023) 135:121–35. doi: 10.1152/japplphysiol.00114.2023, PMID: 37262102

[128] Agüera Sánchez MÁ Barbancho Ma MÁ García-Casares N. Effect of physical exercise on Alzheimer’s disease. A sistematic review. Aten Primaria. (2020) 52:307–18. doi: 10.1016/j.aprim.2018.09.010, PMID: 31153668 PMC7231856

[129] Baranowski BJ Hayward GC Marko DM Mac Pherson REK. Examination of BDNF treatment on BACE1 activity and acute exercise on brain BDNF Signaling. Front Cell Neurosci. (2021) 15:665867. doi: 10.3389/fncel.2021.665867, PMID: 34017238 PMC8129185

[130] Bakhshi K Chance SA. The neuropathology of schizophrenia: a selective review of past studies and emerging themes in brain structure and cytoarchitecture. Neuroscience. (2015) 303:82–102. doi: 10.1016/j.neuroscience.2015.06.028, PMID: 26116523

[131] Olabi B Ellison-Wright I McIntosh AM Wood SJ Bullmore E Lawrie SM. Are there progressive brain changes in schizophrenia? A meta-analysis of structural magnetic resonance imaging studies. Biol Psychiatry. (2011) 70:88–96. doi: 10.1016/j.biopsych.2011.01.032, PMID: 21457946

[132] Czepielewski LS Wang L Gama CS Barch DM. The relationship of intellectual functioning and cognitive performance to brain structure in schizophrenia. Schizophrenia Bull. 43:355–64. Available at: https://psycnet.apa.org/record/2017-31077-020 10.1093/schbul/sbw090 PMC5605271 27369471

[133] Lui S Deng W Huang X Jiang L Ma X Chen H. Association of Cerebral Deficits with Clinical Symptoms in antipsychotic-naive first-episode schizophrenia: An optimized voxel-based Morphometry and resting state functional connectivity study. Am J Psychiatry. (2009) 166:196–205. doi: 10.1176/appi.ajp.2008.08020183, PMID: 18981063

[134] Chen SL Lee SY Chang YH Chen SH Chu CH Wang TY. The BDNF Val 66Met polymorphism and plasma brain-derived neurotrophic factor levels in Han Chinese patients with bipolar disorder and schizophrenia. Prog Neuro-Psychopharmacol Biol Psychiatry. (2014) 51:99–104. doi: 10.1016/j.pnpbp.2014.01.012, PMID: 24468644 PMC7137229

[135] Zhang XY Chen DC Xiu MH Haile CN Luo X Xu K. Cognitive and serum BDNF correlates of BDNF Val 66Met gene polymorphism in patients with schizophrenia and normal controls. Hum Genet. (2012) 131:1187–95. doi: 10.1007/s00439-012-1150-x, PMID: 22362486 PMC3671849

[136] Ray MT Weickert CS Wyatt E Webster MJ. Decreased BDNF, trk B-TK+ and GAD67 mRNA expression in the hippocampus of individuals with schizophrenia and mood disorders. J Psychiatry Neurosci. (2011) 36:195–203. doi: 10.1503/jpn.100048, PMID: 21223646 PMC3080515

[137] Szeszko PR Lipsky R Mentschel C Robinson D Gunduz-Bruce H Sevy S. Brain-derived neurotrophic factor val 66met polymorphism and volume of the hippocampal formation. Mol Psychiatry. (2005) 10:631–6. doi: 10.1038/sj.mp.4001656, PMID: 15768049

[138] Ho BC Andreasen NC Dawson JD Wassink TH. Association between brain-derived neurotrophic factor Val 66Met gene polymorphism and progressive brain volume changes in schizophrenia. Am J Psychiatry. (2007) 164:1890–9. doi: 10.1176/appi.ajp.2007.05111903, PMID: 18056245 PMC3062255

[139] Ho BC Milev P O’Leary DS Librant A Andreasen NC Wassink TH. Cognitive and MRI brain morphometric correlates of brain-derived Neurotrophic factor Val 66Met gene polymorphism in schizophrenia and healthy volunteers. Arch Gen Psychiatry. (2006) 63:731–40. doi: 10.1001/archpsyc.63.7.731, PMID: 16818862 PMC3065118

[140] Zakharyan R Boyajyan A. Brain-derived neurotrophic factor blood levels are decreased in schizophrenia patients and associate with rs 6265 genotypes. Clin Biochem. (2014) 47:1052–5. doi: 10.1016/j.clinbiochem.2014.03.021, PMID: 24713399

[141] Aas M Haukvik UK Djurovic S Tesli M Athanasiu L Bjella T. Interplay between childhood trauma and BDNF val 66met variants on blood BDNF mRNA levels and on hippocampus subfields volumes in schizophrenia spectrum and bipolar disorders. J Psychiatr Res. (2014) 59:14–21. doi: 10.1016/j.jpsychires.2014.08.011, PMID: 25246365

[142] Suárez-Pinilla P Roiz-Santiáñez R de la Foz VOG Mata I Fañanas L Brambilla P. BDNF Val 66Met variants and brain volume changes in non-affective psychosis patients and healthy controls: a 3 year follow-up study. Prog Neuro-Psychopharmacol Biol Psychiatry. (2013) 45:201–6. doi: 10.1016/j.pnpbp.2013.05.014, PMID: 23748016

[143] Harrisberger F Spalek K Smieskova R Schmidt A Coynel D Milnik A. The association of the BDNF Val 66Met polymorphism and the hippocampal volumes in healthy humans: a joint meta-analysis of published and new data. Neurosci Biobehav Rev. (2014) 42:267–78. doi: 10.1016/j.neubiorev.2014.03.011, PMID: 24674929

[144] Molendijk ML Bus BAA Spinhoven P Kaimatzoglou A Voshaar RCO Penninx BWJH. A systematic review and meta-analysis on the association between BDNF val (66) met and hippocampal volume--a genuine effect or a winners curse? Am J Med Genet B Neuropsychiatr Genet. (2012) 159B:731–40. doi: 10.1002/ajmg.b.32078, PMID: 22815222

[145] Ahmed AO Kramer S Hofman N Flynn J Hansen M Martin V. A meta-analysis of brain-derived Neurotrophic factor effects on brain volume in schizophrenia: genotype and serum levels. Neuropsychobiology. (2021) 80:411–24. doi: 10.1159/000514126, PMID: 33706323 PMC8619762

[146] Amato A Ragonese P Ingoglia S Schiera G Schirò G Di Liegro CM. Lactate threshold training program on patients with multiple sclerosis: a multidisciplinary approach. Nutrients. (2021) 13:4284. doi: 10.3390/nu13124284, PMID: 34959834 PMC8704660

[147] Maiworm M. The relevance of BDNF for neuroprotection and neuroplasticity in multiple sclerosis. Front Neurol. (2024) 15:1385042. doi: 10.3389/fneur.2024.1385042 39148705 PMC11325594

[148] Khorshid Ahmad T Acosta C Cortes C Lakowski TM Gangadaran S Namaka M. Transcriptional regulation of brain-derived Neurotrophic factor (BDNF) by methyl CpG binding protein 2 (MeCP2): a novel mechanism for re-myelination and/or myelin repair involved in the treatment of multiple sclerosis (MS). Mol Neurobiol. (2016) 53:1092–107. doi: 10.1007/s12035-014-9074-1, PMID: 25579386

[149] Portaccio E Bellinvia A Prestipino E Nacmias B Bagnoli S Razzolini L. The brain-derived Neurotrophic factor Val 66Met polymorphism can protect against cognitive impairment in multiple sclerosis. Front Neurol. (2021) 12:12. doi: 10.3389/fneur.2021.645220, PMID: 33815257 PMC8011315

[150] Nociti V Romozzi M. The role of BDNF in multiple sclerosis Neuroinflammation. Int J Mol Sci. (2023) 24:8447. doi: 10.3390/ijms24098447, PMID: 37176155 PMC10178984

[151] Lee R Mason A. Cost of aging. Finance Dev. (2017) 54:7–9. PMID: 28835725 PMC5564373

[152] WHO. Towards a dementia plan: a WHO guide [Internet]; (2018). Available at: https://www.who.int/publications/i/item/9789241514132 (Accessed May 24, 2018).

[153] Tisher A Salardini A. A comprehensive update on treatment of dementia. Semin Neurol. (2019) 39:167–78. doi: 10.1055/s-0039-1683408, PMID: 30925610

[154] Sharma K. Cholinesterase inhibitors as Alzheimer’s therapeutics (review). Mol Med Rep. (2019) 20:1479–87. doi: 10.3892/mmr.2019.10374, PMID: 31257471 PMC6625431

[155] Birks JS Harvey RJ. Donepezil for dementia due to Alzheimer’s disease. Cochrane Database Syst Rev. (2018) 6:1–235. doi: 10.1002/14651858.CD001190.pub3, PMID: 29923184 PMC6513124

[156] Shah H Albanese E Duggan C Rudan I Langa KM Carrillo MC. Research priorities to reduce the global burden of dementia by 2025. Lancet Neurol. (2016) 15:1285–94. doi: 10.1016/S1474-4422(16)30235-6, PMID: 27751558

[157] Vidoni ED Gayed MR Honea RA Savage CR Hobbs D Burns JM. Alzheimer disease alters the relationship of cardiorespiratory fitness with brain activity during the Stroop task. Phys Ther. (2013) 93:993–1002. doi: 10.2522/ptj.20120465, PMID: 23559521 PMC3704231

[158] Liang JH Xu Y Lin L Jia RX Zhang HB Hang L. Comparison of multiple interventions for older adults with Alzheimer disease or mild cognitive impairment: a PRISMA-compliant network meta-analysis. Medicine. (2018) 97:e10744. doi: 10.1097/MD.0000000000010744, PMID: 29768349 PMC5976284

[159] Vidoni ED Honea RA Billinger SA Swerdlow RH Burns JM. Cardiorespiratory fitness is associated with atrophy in Alzheimer’s and aging over 2 years. Neurobiol Aging. (2012) 33:1624–32. doi: 10.1016/j.neurobiolaging.2011.03.016, PMID: 21531480 PMC3156963

[160] Parrini M Ghezzi D Deidda G Medrihan L Castroflorio E Alberti M. Aerobic exercise and a BDNF-mimetic therapy rescue learning and memory in a mouse model of Down syndrome. Sci Rep. (2017) 7:16825. doi: 10.1038/s41598-017-17201-8, PMID: 29203796 PMC5715062

[161] Woodard JL Nielson KA Sugarman MA Smith JC Seidenberg M Durgerian S. Lifestyle and genetic contributions to cognitive decline and hippocampal integrity in healthy aging. Curr Alzheimer Res. (2012) 9:436–46. doi: 10.2174/156720512800492477, PMID: 22272622 PMC3584642

[162] Valenzuela PL Castillo-García A Morales JS de la Villa P Hampel H Emanuele E. Exercise benefits on Alzheimer’s disease: state-of-the-science. Ageing Res Rev. (2020) 62:101108. doi: 10.1016/j.arr.2020.101108, PMID: 32561386

[163] Venegas-Sanabria LC Cavero-Redondo I Martínez-Vizcaino V Cano-Gutierrez CA Álvarez-Bueno C. Effect of multicomponent exercise in cognitive impairment: a systematic review and meta-analysis. BMC Geriatr. (2022) 22:1–11. doi: 10.1186/s12877-022-03302-1, PMID: 35879665 PMC9316334

[164] Szuhany KL Bugatti M Otto MW. A meta-analytic review of the effects of exercise on brain-derived neurotrophic factor. J Psychiatr Res. (2015) 60:56–64. doi: 10.1016/j.jpsychires.2014.10.003, PMID: 25455510 PMC4314337

[165] Marinus N Hansen D Feys P Meesen R Timmermans A Spildooren J. The impact of different types of exercise training on peripheral blood brain-derived Neurotrophic factor concentrations in older adults: a meta-analysis. Sports Med. (2019) 49:1529–46. doi: 10.1007/s40279-019-01148-z, PMID: 31270754

[166] Raji CA Merrill DA Eyre H Mallam S Torosyan N Erickson KI. Longitudinal relationships between caloric expenditure and Gray matter in the cardiovascular health study. J Alzheimers Dis. (2016) 52:719–29. doi: 10.3233/JAD-160057, PMID: 26967227 PMC4927887

[167] Honea RA Thomas GP Harsha A Anderson HS Donnelly JE Brooks WM. Cardiorespiratory fitness and preserved medial temporal lobe volume in Alzheimer’s disease. Alzheimer Dis Assoc Disord. (2009) 23:188–97. doi: 10.1097/WAD.0b013e31819cb8a2, PMID: 19812458 PMC2760037

[168] Boyle CP Raji CA Erickson KI Lopez OL Becker JT Gach HM. Physical activity, body mass index, and brain atrophy in Alzheimer’s disease. Neurobiol Aging. (2015) 36:S194–202. doi: 10.1016/j.neurobiolaging.2014.05.036, PMID: 25248607 PMC4303036

[169] Mackay CP Kuys SS Brauer SG. The effect of aerobic exercise on brain-derived Neurotrophic factor in people with neurological disorders: a systematic review and meta-analysis. Neural Plast. (2017) 2017:1–9. doi: 10.1155/2017/4716197, PMID: 29057125 PMC5625797

[170] Braskie MN Boyle CP Rajagopalan P Gutman BA Toga AW Raji CA. Physical activity, inflammation, and volume of the aging brain. Neuroscience. (2014) 273:199–209. doi: 10.1016/j.neuroscience.2014.05.005, PMID: 24836855 PMC4076831

[171] Schultz SA Boots EA Almeida RP Oh JM Einerson J Korcarz CE. Cardiorespiratory fitness attenuates the influence of amyloid on cognition. J Int Neuropsychol Soc. (2015) 21:841–50. doi: 10.1017/S1355617715000843, PMID: 26581795 PMC4716656

[172] Tan ZS Spartano NL Beiser AS DeCarli C Auerbach SH Vasan RS. Physical activity, brain volume, and dementia risk: the Framingham study. J Gerontol A Biol Sci Med Sci. (2017) 72:789–95. doi: 10.1093/gerona/glw130, PMID: 27422439 PMC6075525

[173] Nagahara AH Merrill DA Coppola G Tsukada S Schroeder BE Shaked GM. Neuroprotective effects of brain-derived neurotrophic factor in rodent and primate models of Alzheimer’s disease. Nat Med. (2009) 15:331–7. doi: 10.1038/nm.1912, PMID: 19198615 PMC2838375

[174] Dadkhah M Saadat M Ghorbanpour AM Moradikor N. Experimental and clinical evidence of physical exercise on BDNF and cognitive function: a comprehensive review from molecular basis to therapy. Brain Behav Immun Integr. (2023) 3:100017. doi: 10.1016/j.bbii.2023.100017

[175] Antunes BM Rossi FE Teixeira AM Lira FS. Short-time high-intensity exercise increases peripheral BDNF in a physical fitness-dependent way in healthy men. Eur J Sport Sci. (2020) 20:43–50. doi: 10.1080/17461391.2019.1611929, PMID: 31057094

[176] García-Suárez PC Rentería I Moncada-Jiménez J Fry AC Jiménez-Maldonado A. Acute systemic response of BDNF, lactate and cortisol to strenuous exercise modalities in healthy untrained women. Dose-Response. (2020) 18:1559325820970818. doi: 10.1177/1559325820970818, PMID: 33354170 PMC7734519

[177] Rasmussen P Brassard P Adser H Pedersen MV Leick L Hart E. Evidence for a release of brain-derived neurotrophic factor from the brain during exercise. Exp Physiol. (2009) 94:1062–9. doi: 10.1113/expphysiol.2009.048512, PMID: 19666694

[178] El-Sayes J Harasym D Turco CV Locke MB Nelson AJ. Exercise-induced neuroplasticity: a mechanistic model and prospects for promoting plasticity. Neuroscientist. (2019) 25:65–85. doi: 10.1177/1073858418771538, PMID: 29683026

[179] Huang T Larsen KT Ried-Larsen M Møller NC Andersen LB. The effects of physical activity and exercise on brain-derived neurotrophic factor in healthy humans: a review. Scand J Med Sci Sports. (2014) 24:1–10. doi: 10.1111/sms.12069, PMID: 23600729

[180] Ribeiro D Petrigna L Pereira FC Muscella A Bianco A Tavares P. The impact of physical exercise on the circulating levels of BDNF and NT 4/5: a review. Int J Mol Sci. (2021) 22:8814. doi: 10.3390/ijms22168814, PMID: 34445512 PMC8396229

[181] Ross RE Saladin ME George MS Gregory CM. High-intensity aerobic exercise acutely increases brain-derived Neurotrophic factor. Med Sci Sports Exerc. (2019) 51:1698–709. doi: 10.1249/MSS.0000000000001969, PMID: 30829963 PMC6629513

[182] Wang Y Zhou H Luo Q Cui S. The effect of physical exercise on circulating brain-derived neurotrophic factor in healthy subjects: a meta-analysis of randomized controlled trials. Brain Behav. (2022) 12:e2544. doi: 10.1002/brb3.2544, PMID: 35274832 PMC9014996

[183] Trombetta IC DeMoura JR Alves CR Carbonari-Brito R Cepeda FX Lemos JR. Níveis Séricos do BDNF na Proteção Cardiovascular e em Resposta ao Exercício. Arq Bras Cardiol. (2020) 115:263–9. doi: 10.36660/abc.20190368, PMID: 32876194 PMC8384297

[184] Arazi H Babaei P Moghimi M Asadi A. Acute effects of strength and endurance exercise on serum BDNF and IGF-1 levels in older men. BMC Geriatr. (2021) 21:50. doi: 10.1186/s12877-020-01937-6, PMID: 33441099 PMC7807435

[185] Kim K Sung YH Seo JH Lee SW Lim BV Lee CY. Effects of treadmill exercise-intensity on short-term memory in the rats born of the lipopolysaccharide-exposed maternal rats. J Exerc Rehabil. (2015) 11:296–302. doi: 10.12965/jer.150264, PMID: 26730379 PMC4697777

[186] Nay K Smiles WJ Kaiser J McAloon LM Loh K Galic S. Molecular mechanisms underlying the beneficial effects of exercise on brain function and neurological disorders. Int J Mol Sci. (2021) 22:4052. doi: 10.3390/ijms22084052, PMID: 33919972 PMC8070923

[187] Vaynman S Ying Z Gomez-Pinilla F. Hippocampal BDNF mediates the efficacy of exercise on synaptic plasticity and cognition. Eur J Neurosci. (2004) 20:2580–90. doi: 10.1111/j.1460-9568.2004.03720.x, PMID: 15548201

[188] Pluchino N Cubeddu A Begliuomini S Merlini S Giannini A Bucci F. Daily variation of brain-derived neurotrophic factor and cortisol in women with normal menstrual cycles, undergoing oral contraception and in postmenopause. Hum Reprod. (2009) 24:2303–9. doi: 10.1093/humrep/dep119, PMID: 19491202

[189] Bugge Kambestad O Sirevåg K Mrdalj J Hovland A Bruun Endal T Andersson E. Physical exercise and serum BDNF levels: accounting for the Val 66Met polymorphism in older adults. Cogn Behav Neurol. (2023) 36:219–27. doi: 10.1097/WNN.0000000000000349, PMID: 37404130 PMC10683974

[190] Rentería I García-Suárez PC Martínez-Corona DO Moncada-Jiménez J Plaisance EP Jimé N-MA. Short-term high-intensity interval training increases systemic brain-derived neurotrophic factor (BDNF) in healthy women. Eur J Sport Sci. (2020) 20:516–24. doi: 10.1080/17461391.2019.1650120, PMID: 31386821

[191] Carek PJ Laibstain SE Carek SM. Exercise for the treatment of depression and anxiety. Int J Psychiatry Med. (2011) 41:15–28. doi: 10.2190/PM.41.1.c, PMID: 21495519

[192] Vivar C Potter MC van Praag H. All about running: synaptic plasticity, growth factors and adult hippocampal neurogenesis. Curr Top Behav Neurosci. (2012) 15:189–210. doi: 10.1007/7854_2012_220, PMID: 22847651 PMC4565722

[193] Fletcher JL Wood RJ Nguyen J Norman EML Jun CMK Prawdiuk AR. Targeting Trk B with a brain-derived Neurotrophic factor mimetic promotes myelin repair in the brain. J Neurosci. (2018) 38:7088–99. doi: 10.1523/JNEUROSCI.0487-18.2018, PMID: 29976621 PMC6596092

[194] Callaghan CK Rouine J O’Mara SM. Exercise prevents IFN-α-induced mood and cognitive dysfunction and increases BDNF expression in the rat. Physiol Behav. (2017) 179:377–83. doi: 10.1016/j.physbeh.2017.07.018, PMID: 28711394

[195] Bastioli G Arnold JC Mancini M Mar AC Gamallo-Lana B Saadipour K. Voluntary exercise boosts striatal dopamine release: evidence for the necessary and sufficient role of BDNF. J Neurosci. (2022) 42:4725–36. doi: 10.1523/JNEUROSCI.2273-21.2022, PMID: 35577554 PMC9186798

[196] Piotrowicz Z Chalimoniuk M Płoszczyca K Czuba M Langfort J. Exercise-induced elevated BDNF level does not prevent cognitive impairment due to acute exposure to moderate hypoxia in well-trained athletes. Int J Mol Sci. (2020) 21:5569. doi: 10.3390/ijms21155569, PMID: 32759658 PMC7432544

[197] Liu T Li H Colton JP Ge S Li C. The BDNF Val 66Met polymorphism, regular exercise, and cognition: a systematic review. West J Nurs Res. (2020) 42:660–73. doi: 10.1177/0193945920907308, PMID: 32075548

[198] Li G Peskind ER Millard SP Chi P Sokal I Yu CE. Cerebrospinal fluid concentration of brain-derived Neurotrophic factor and cognitive function in non-demented subjects. PLoS One. (2009) 4:e5424. doi: 10.1371/journal.pone.0005424, PMID: 19412541 PMC2671606

[199] Sun B Peng AS Liu PJ Wang MJ Ding HL Hu YS. Neuroprotection of exercise: P2X4R and P2X7R regulate BDNF actions. Purinergic Signal. (2023) 19:297–303. doi: 10.1007/s11302-022-09879-x, PMID: 35821455 PMC9275535

[200] Kennedy G Hardman RJ Macpherson H Scholey AB Pipingas A. How does exercise reduce the rate of age-associated cognitive decline? A review of potential mechanisms. J Alzheimers Dis. (2016) 55:1–18. doi: 10.3233/JAD-160665, PMID: 27636853

[201] Tsai CL Pan CY Tseng YT Chen FC Chang YC Wang TC. Acute effects of high-intensity interval training and moderate-intensity continuous exercise on BDNF and irisin levels and neurocognitive performance in late middle-aged and older adults. Behav Brain Res. (2021) 413:113472. doi: 10.1016/j.bbr.2021.113472, PMID: 34274372

[202] Bus BAA Tendolkar I Franke B de Graaf J Heijer MD Buitelaar JK. Serum brain-derived neurotrophic factor: determinants and relationship with depressive symptoms in a community population of middle-aged and elderly people. World J Biol Psychiatry. (2012) 13:39–47. doi: 10.3109/15622975.2010.545187, PMID: 21247257 PMC3279138

[203] Ziegenhorn AA Schulte-Herbrüggen O Danker-Hopfe H Malbranc M Hartung HD Anders D. Serum neurotrophins—a study on the time course and influencing factors in a large old age sample. Neurobiol Aging. (2007) 28:1436–45. doi: 10.1016/j.neurobiolaging.2006.06.011, PMID: 16879899

[204] Shimada H Makizako H Doi T Yoshida D Tsutsumimoto K Anan Y. A large, cross-sectional observational study of serum BDNF, cognitive function, and mild cognitive impairment in the elderly. Front Aging Neurosci. (2014) 6:1–9. doi: 10.3389/fnagi.2014.00069, PMID: 24782766 PMC3995061

[205] Huang X Wang J Zhang S Zhao X An R Lan Y. Plasma BDNF/Irisin ratio associates with cognitive function in older people. J Alzheimers Dis. (2024) 99:1261–71. doi: 10.3233/JAD-231347, PMID: 38788070

[206] Hirsch MA van Wegen EE Newman MA Heyn PC. Exercise-induced increase in brain-derived neurotrophic factor in human Parkinson’s disease: a systematic review and meta-analysis. Transl Neurodegener. (2018) 7:1–12. 29568518 10.1186/s40035-018-0112-1 PMC5859548

